# Gut Analysis Toolbox – automating quantitative analysis of enteric neurons

**DOI:** 10.1242/jcs.261950

**Published:** 2024-10-30

**Authors:** Luke Sorensen, Adam Humenick, Sabrina S. B. Poon, Myat Noe Han, Narges S. Mahdavian, Matthew C. Rowe, Ryan Hamnett, Estibaliz Gómez-de-Mariscal, Peter H. Neckel, Ayame Saito, Keith Mutunduwe, Christie Glennan, Robert Haase, Rachel M. McQuade, Jaime P. P. Foong, Simon J. H. Brookes, Julia A. Kaltschmidt, Arrate Muñoz-Barrutia, Sebastian K. King, Nicholas A. Veldhuis, Simona E. Carbone, Daniel P. Poole, Pradeep Rajasekhar

**Affiliations:** ^1^ Drug Discovery Biology, Monash Institute of Pharmaceutical Sciences, Monash University, Parkville, VIC 3052, Australia; ^2^ Flinders Health and Medical Research Institute, College of Medicine and Public Health, Flinders University, Bedford Park, SA 5042, Australia; ^3^ Department of Anatomy and Physiology, The University of Melbourne, Parkville, VIC 3052, Australia; ^4^ Wu Tsai Neurosciences Institute, Stanford University, Stanford, CA 94305, USA; ^5^ Department of Neurosurgery, Stanford University School of Medicine, Stanford, CA 94305, USA; ^6^ Optical Cell Biology Group, Instituto Gulbenkian de Ciência, Oeiras 2780-156, Portugal; ^7^ Institute of Clinical Anatomy and Cell Analysis, University of Tübingen, Tübingen 72076, Germany; ^8^ Center for Scalable Data Analytics and Artificial Intelligence (ScaDS.AI) Dresden/Leipzig, Universität Leipzig, Humboldtstraße 25, Leipzig 04105, Germany; ^9^ Gut Barrier and Disease Laboratory, Department of Anatomy and Physiology, The University of Melbourne, Melbourne, VIC 3010, Australia; ^10^ Department of Medicine, Western Health, The University of Melbourne, Melbourne, VIC 3021, Australia; ^11^ Australian Institute for Musculoskeletal Science (AIMSS), The University of Melbourne, Melbourne, VIC 3021, Australia; ^12^ Bioengineering Department, Universidad Carlos III de Madrid, ES 28911, Leganés, Spain; ^13^ Bioengineering Division, Instituto de Investigación Sanitaria Gregorio Marañon, ES 28007, Madrid, Spain; ^14^ Department of Paediatric Surgery, The Royal Children's Hospital, Parkville, VIC 3052, Australia; ^15^ Surgical Research, Murdoch Children's Research Institute, Parkville, VIC 3052, Australia; ^16^ Department of Paediatrics, The University of Melbourne, Parkville, VIC 3010, Australia; ^17^ Centre for Dynamic Imaging, The Walter and Eliza Hall Institute of Medical Research, Parkville, VIC 3052, Australia; ^18^ Department of Medical Biology, The University of Melbourne, Parkville, VIC 3052, Australia

**Keywords:** Enteric nervous system, Gut analysis toolbox, Image analysis, Machine learning, Fiji, Spatial analysis

## Abstract

The enteric nervous system (ENS) consists of an extensive network of neurons and glial cells embedded within the wall of the gastrointestinal (GI) tract. Alterations in neuronal distribution and function are strongly associated with GI dysfunction. Current methods for assessing neuronal distribution suffer from undersampling, partly due to challenges associated with imaging and analyzing large tissue areas, and operator bias due to manual analysis. We present the Gut Analysis Toolbox (GAT), an image analysis tool designed for characterization of enteric neurons and their neurochemical coding using two-dimensional images of GI wholemount preparations. GAT is developed in Fiji, has a user-friendly interface, and offers rapid and accurate segmentation via custom deep learning (DL)-based cell segmentation models developed using StarDist, as well as a ganglia segmentation model in deepImageJ. We apply proximal neighbor-based spatial analysis to reveal differences in cellular distribution across gut regions using a public dataset. In summary, GAT provides an easy-to-use toolbox to streamline routine image analysis tasks in ENS research. GAT enhances throughput, allowing rapid unbiased analysis of larger tissue areas, multiple neuronal markers and numerous samples.

## Teaser

**Summary:** Gut Analysis Toolbox is a user-friendly image analysis tool for rapid and accurate characterization of neuronal distribution in 2D images of the gut wall, using custom-trained deep learning models.

## INTRODUCTION

The enteric nervous system (ENS) is a network of neurons and glial cells located within the wall of the gastrointestinal (GI) tract. The ENS extends along the esophagus to the rectum and is estimated to comprise ∼168 million neurons, which is comparable to the number of neurons in the spinal cords of humans, mice and guinea pigs (Michel et al., 2022). It is critical for the regulation of secretion, absorption and immune function, and for coordination of gut motility (Furness, 2012). The absence or loss of enteric neurons results in GI dysfunction, as evidenced in enteric neuropathies such as Hirschsprung disease, achalasia and Chagas disease (Burns et al., 2016; Heuckeroth, 2018; Schäppi et al., 2013; Vaezi et al., 2016). Alterations to specific enteric neuron populations that express distinct combinations of neuropeptides, enzymes or neurochemicals are also evident in other diseases that impact gut function. These include inflammatory bowel disease (Brierley and Linden, 2014), diabetes (Chandrasekharan et al., 2011; Demedts et al., 2013) and Alzheimer's disease (Niesler et al., 2021; Semar et al., 2013; Van Ginneken et al., 2011). Researchers use enteric neuronal counts as a key metric to describe any neurochemical changes in these diseases. Typically, this is achieved by manually counting cells in small intestinal segments or, more recently, via semi-automated methods (Cairns et al., 2021; Cavin et al., 2023; Kapur, 2013; Kobayashi et al., 2021; Nestor-Kalinoski et al., 2022; Parker et al., 2022; Schäppi et al., 2013). These cell counts from localized areas within a specimen are then used to make inferences about broader changes to the bowel region being studied and any changes associated with disease. However, the estimated number of cells counted can be affected by: (1) the tissue preparation examined, as cell density estimates using tissue sections can be prone to sampling and operator bias compared to the use of wholemount preparations (Kapur, 2013; Swaminathan and Kapur, 2010); (2) the age of the animal (Gamage et al., 2013); (3) the tissue region examined, due to regional differences in the distribution of ENS circuitry (Hamnett et al., 2022b; Nestor-Kalinoski et al., 2022); (4) the number of tissue specimens and locations sampled (Nestor-Kalinoski et al., 2022); and (5) operator bias during the tissue preparation, sampling or manual counting steps (Kapur, 2013; Schäppi et al., 2013).

The major limitation of current approaches for neuronal quantification in large tissue specimens is the use of manual cell counting. This process is slow, labor intensive and prone to inter-observer variability. In our opinion, the continued use of manual counting processes is largely due to the lack of easy-to-use, ENS-specific image analysis software.

The need for image analysis software in neurogastroenterology is evidenced by the increasing number of image analysis workflows and tools that have become available in recent years, such as COUNTEN and the use of machine learning approaches in Fiji (Cairns et al., 2021; Kobayashi et al., 2021). However, to use these workflows computational expertise is required, and the software parameters need to be optimized for new datasets. COUNTEN is highly dependent on images of robust and homogeneous staining, which is not always achievable for all intestinal preparations (Kobayashi et al., 2021). The image quality affects the ability of image analysis techniques to accurately detect cells. This could be due to multiple factors, such as (1) poor quality of dissection of intestinal layers, which can affect antibody penetration and labeling of the cells in the ENS; (2) specificity of the antibodies, markers and fluorophores used during staining; and (3) variations in sample preparation. Furthermore, image acquisition specifications, including the bit depth and dynamic range, can significantly affect downstream image analysis. All these factors can pose challenges to the widespread adoption of such software, necessitating the development of customized analytical workflows for each use case.

**Fig. 1.**
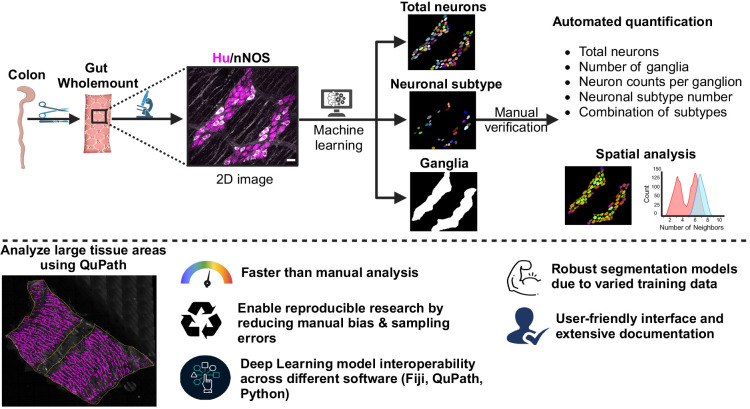
**Overview of the GAT workflow.** Top: GAT can segment neurons, neurons expressing neurochemical markers, and ganglia in fluorescently labeled 2D images using pre-trained DL models. GAT allows manual verification of the segmentation, followed by automated quantification of cell counts. The cellular distribution can be subsequently quantified via proximal neighbor analysis. Bottom: large 2D images can be analyzed in QuPath using the GAT DL models. Extensive documentation and videos on how to use GAT are available at https://gut-analysis-toolbox.gitbook.io/docs/. Myenteric wholemount images were either acquired as part of the study reported by DiCello et al. (2020) (top) or are reproduced from Kalinoski and Howard (2021) (bottom; reproduced under the terms of a CDLA-Permissive-1.0 license). Scale bar: 30 µm. Created in BioRender (https://BioRender.com/q94f538).

We have developed the Gut Analysis Toolbox (GAT) for the Fiji distribution of ImageJ (Schindelin et al., 2012). GAT can be used to analyze and quantify cells within the ENS. GAT uses deep learning (DL)-based cell segmentation models developed with StarDist (Schmidt et al., 2018) for segmenting enteric neurons and neuronal subtypes. A pre-trained TensorFlow model was used to segment ganglia, and this model is accessible in Fiji using deepImageJ (Gómez-de-Mariscal et al., 2021). These models are integrated into GAT for rapid and reproducible quantification of key metrics such as total neuronal counts, neurochemical marker distribution and cell number per ganglion (Fig. 1). The DL models were trained on manually annotated data from mouse, rat and human colon wholemount preparations to ensure that they can effectively segment a wide variety of images (Chen et al., 2023; DiCello et al., 2020; Graham et al., 2020b; Nestor-Kalinoski et al., 2022). The training images were acquired using confocal and widefield microscopes from different research groups. Proximal neighbor analysis was used to characterize neuronal distribution using CLIJ (Haase et al., 2020a preprint, 2020b). Comprehensive installation and usage instructions, along with sample data and tutorial videos, can be found in the documentation available at https://gut-analysis-toolbox.gitbook.io/docs/. The GAT workflow is also usable from within the QuPath software to enable analysis of large two-dimensional (2D) images (Bankhead et al., 2017). The enhanced throughput of GAT facilitates sampling and analysis of larger tissue areas, thus minimizing potential sampling errors and biases.

## RESULTS

### Development and benchmarking of the DL models

To develop effective DL models, it is essential to have diverse datasets that encompass the inherent variability in images from various sources. To meet this need, we collected ENS images from four different research laboratories, which were acquired as part of previously published studies (Chen et al., 2023; DiCello et al., 2020; McQuade et al., 2021; Poon et al., 2022), and from two publicly available datasets (Graham et al., 2020a; Kalinoski and Howard, 2021). The collected images had been captured using a variety of microscopes, and the labeled tissues originated from different animal species, including mice, humans and rats, as detailed in Table S1. The details for images with pan-neuronal marker Hu (herein referring to HuC and/or HuD, also known as ELAVL3 and ELAVL4, respectively) are summarized in Table S1. For the enteric neuron subtype model (Table S2), nine neurochemical markers were used, with 42% of the images representing neuronal NOS (nNOS, also known as NOS1). The ganglia model was trained using various neuronal markers in combination with Hu, as listed in Table S3. The effects of inadequate sampling on enteric neuron counts were also tested (Fig. S1).

StarDist (Schmidt et al., 2018) was used to train DL models for segmenting all enteric neurons (Fig. S2A,B). A UNet architecture was utilized for the ganglia model (Ronneberger et al., 2015). The training and evaluation of the models were conducted using ZeroCostDL4Mic (von Chamier et al., 2021). Further details on the curation of training data and software versions used can be found in the Materials and Methods section. The ZeroCostDL4Mic Google Colab notebooks used for training and quality control are available online (Sorensen et al., 2022; https://doi.org/10.5281/zenodo.10460434).

**Fig. 2.**
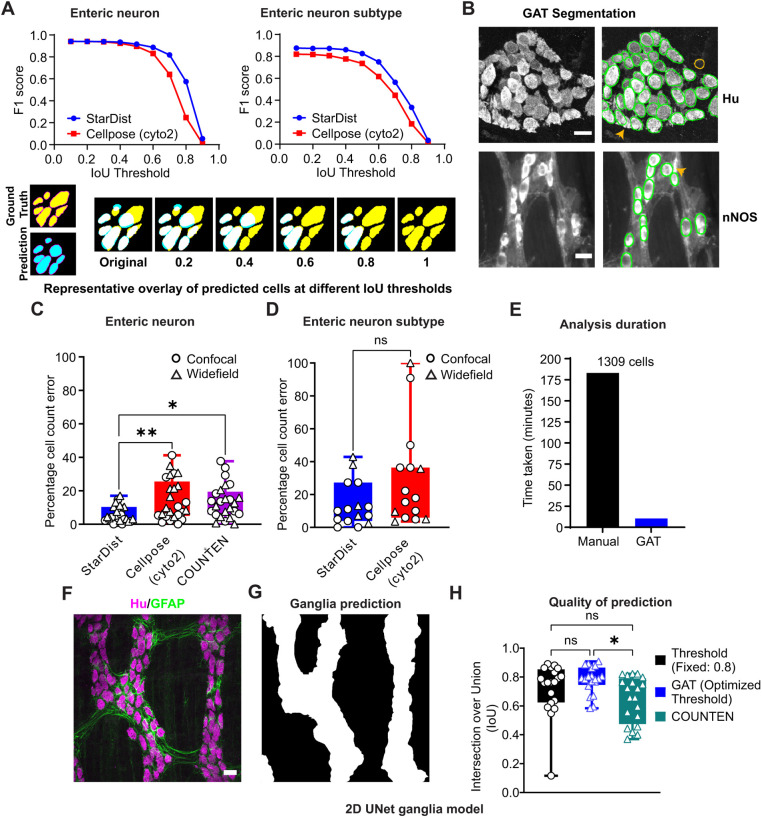
**GAT segments neurons and ganglia with high accuracy.** (A) Segmentation results for Hu-labeled enteric neurons (mouse, rat and human) obtained using the GAT StarDist model (Table S1) and Cellpose (cyto2 model) have comparable F1 scores (top left). However, the GAT StarDist model for other neurochemical markers (Table S2) has a better F1 score at various IoU thresholds (top right). Bottom: representative images of ground truth cells (yellow) and predicted cells (cyan) are overlaid to visualize how more accurate predictions are retained at higher IoU thresholds (white indicates overlap between the ground truth and predicted images). Generally, the more predictions that are retained at higher IoU thresholds, the better the model (some cells in the prediction image were manually edited to illustrate the changes at higher IoU thresholds). Data shown are from test datasets with 3830 neurons, 25 images for the enteric neuron model, and with 359 neurons, 15 images for the enteric neuron subtype model. (B) The StarDist model can be used to detect bright cells, dim cells and overlapping cells. The representative images of GAT StarDist model segmentation depict mouse enteric neurons that were either labeled with Hu and segmented using the enteric neuron model (top), or labeled with nNOS and segmented using the enteric neuron subtype model (bottom). Green outlines highlight GAT-detected cells. The orange outline shows a false-positive cell, and the orange arrowheads indicate a missed cell. Scale bars: 20 µm. A pixel size of 0.5 µm/pixel was used for segmentation. (C) When used to count enteric neurons in the same test dataset as panel A, the lowest percentage of error was achieved using the GAT StarDist model, compared to Cellpose models and COUNTEN (mean±s.d.; 25 images, 3830 cells). *P<0.05; **P<0.01 (one-way ANOVA with Tukey's multiple comparison test). (D) The StarDist enteric neuron subtype model produced a lower percentage error compared to Cellpose when used to count different enteric neurons from the test dataset based on the neurochemical marker expressed (mean±s.d.; 15 images, 359 cells). ns, not significant (*P*=0.07; two-tailed unpaired *t*-test). (E) The total time taken to segment 1309 mouse enteric neurons (8 images) was faster using GAT compared to manual segmentation. (F,G) A 2D UNet model was trained to segment ganglia based on the presence of various markers (Table S3) that label the neuron or glial fibers. The representative images demonstrate prediction and segmentation of ganglia in mouse tissue using Hu and GFAP. Scale bar: 30 µm. (H) When evaluated on a test image dataset (human, rat and mouse), the ganglia segmentation model has a mean IoU of 0.72±0.17 when using a fixed threshold of 0.8, and 0.78±0.09 with a deepImageJ optimized threshold for each image. COUNTEN has a lower IoU of 0.645±0.16 (mean±s.d.; 20 images). *P<0.01; ns, not significant (one-way ANOVA with Tukey's multiple comparisons test).

The performance of the ‘enteric neuron model’ was evaluated on a test dataset and compared to a widely used cell segmentation software, Cellpose (v0.7) (Stringer et al., 2021). Fig. 2A presents the evaluation of object detection accuracy (F1 score) and shape alignment accuracy (intersection over union, IoU) (Caicedo et al., 2019). A higher F1 score at a higher IoU indicates better segmentation performance (Fig. 2A). The Hu segmentation results showed comparable performance between the StarDist and Cellpose (cyto2) models. However, when examining the neuronal subtype models, a rightward shift in F1 scores of the StarDist model indicates a modest improvement when compared to the Cellpose cyto2 model (Fig. 2A). As StarDist approximates the shape of a cell using star-convex polygons, the predicted objects have smoother outlines relative to the original cell shape (Fig. 2B; Fig. S2C,D).

The primary goal of GAT is to estimate cell counts, and this metric is not necessarily captured by the F1 score, which evaluates segmentation quality. The StarDist models approximate cell shape, and this leads to smoother outlines, meaning that the final segmentation result will slightly differ from the ground truth, leading to reduced F1 scores (Fig. S2C,D). The ‘percentage cell count error’, which is defined as:

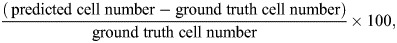
is a more direct measure of the accuracy of these DL models for evaluating cell count, compared to the F1 score. Importantly, this approach allows for an objective comparison with COUNTEN using the default settings recommended by the authors (Kobayashi et al., 2021). In this context, a lower percentage error indicates enhanced performance. The percentage cell count error was estimated using the same test datasets as those used for the F1 score. The StarDist neuron model had significantly better accuracy, with only 6.09±4.8% error in cell counts compared to 14.6±12.1% for Cellpose (25 images, 3830 neurons, mean±s.d.; one-way ANOVA with Tukey's multiple comparison test, *P*=0.0064). The percentage error for COUNTEN (Fig. 2C) was also higher than that for GAT at 13.54±10% (one-way ANOVA with Tukey's multiple comparison test, *P*=0.0195). When testing the accuracy of segmenting enteric neurons expressing the markers calbindin (Calb), calretinin (Calret), nNOS and neurofilament M (NFM, also known as NEFM), the StarDist model demonstrated a similar cell count percentage error (13.8±13.1%) compared to that of Cellpose (29.5±29.3%, mean±s.d.; 359 cells, 15 images; two-tailed unpaired *t*-test, *P*=0.07) (Fig. 2D). Notably, being a generalist cell segmentation algorithm, Cellpose still had high IoU scores on the enteric neuron segmentation task, with higher cell count accuracies for 2D confocal images (Fig. 2C). The Cellpose predictions had higher cell splitting and merging errors (Wolny et al., 2020) compared to the StarDist predictions (Fig. S2E–G). Fine-tuning the settings or training the Cellpose models on these enteric neuron datasets will most likely produce a better performing Cellpose enteric neuronal model (Pachitariu and Stringer, 2022). However, this was not explored due to the lack of a standalone Fiji plugin for Cellpose. The percentage error was also calculated for the VersatileFluo model provided by StarDist and was found to be significantly higher than that for the GAT StarDist model (Fig. S2I; 25 images, 3830 neurons; two-tailed unpaired *t*-test, *P*=0.000015).

Semi-automation using GAT increased analysis throughput. Manual segmentation of 1309 enteric neurons took 183 min in total, across three researchers, whereas segmentation using GAT and the enteric neuron DL model took only 10.4 min by a single person (Fig. 2E).

To enable segmentation of ganglia using GAT, a 2D UNet model was trained using ZeroCostDL4Mic (von Chamier et al., 2021) on images of Hu labeling in combination with a second marker for neuronal or glial fibers (Table S3). The ganglia model was subsequently exported in a format compatible for use within the Fiji deepImageJ plugin (Gómez-de-Mariscal et al., 2021). A limitation is that the postprocessing threshold value applied to the probability map output from deepImageJ can impact the accuracy of the ganglia outlines. To evaluate this, an arbitrary threshold of 0.8 was compared with the default deepImageJ optimized threshold. The GAT ganglia model performed significantly better than COUNTEN (Fig. 2F–H) when measuring the IoU at an optimized threshold (IoU of 0.78±0.09 for GAT versus 0.64±0.16 for COUNTEN; mean±s.d.; one-way ANOVA with Tukey's multiple comparison test, *P*=0.01), which was in contrast to performance using the fixed threshold (IoU of 0.72±0.1; one-way ANOVA with Tukey's multiple comparison test, *P*=0.23). Although there was not a significant difference between IoU for GAT at an optimized threshold versus that at the fixed threshold (one-way ANOVA with Tukey's multiple comparison test, *P*=0.39), the variability in prediction was lower using the optimized threshold. Not all laboratories use markers to label the ganglionic border. In this instance, the neuronal outline can be expanded by a user-specified distance to approximate ganglionic area.

### Proximal neighbor analysis in GAT can objectively detect regional differences in cellular distribution

An accurate estimation of neuronal densities in gut wholemount preparations can be impacted by the degree of stretch applied during tissue processing (Fig. S1A). Nonetheless, the stretch applied does not alter the cellular architecture and spatial relationships between cells. Any change in cell density results in a proportional change in the number of neighbors around each cell. As the spatial relationship is unaffected by tissue stretch, the number of proximal neighbors (PNs) is a robust measure for characterizing cellular distribution. To apply PN measurements in GAT, a threshold distance value was determined to define the distance within which cells were considered neighbors. The edge-to-edge distances between the segmented neurons in the ganglia were measured in images of the myenteric wholemount preparations of the mouse colon (Tables S1 and S2). To define a threshold distance value for considering a cell a PN, the average PN distance between neurons (edge-to-edge distance) in the ganglia was calculated using the ‘Local Thickness’ plugin (Dougherty and Kunzelmann, 2007) in Fiji (Fig. S3). For the myenteric wholemount preparations of the mouse colon, this was determined to be 6.32±5.17 µm (*n*=3643 cells, 130 ganglia; mean±s.d.; *N*=11), which was rounded up to 6.5 µm for use within GAT (Fig. S3C,D). This value is customizable in GAT to account for differences in tissue preparation or varying cell sizes across species. The neighbor count map allowed the examination of any changes associated with different distance values.

**Fig. 3.**
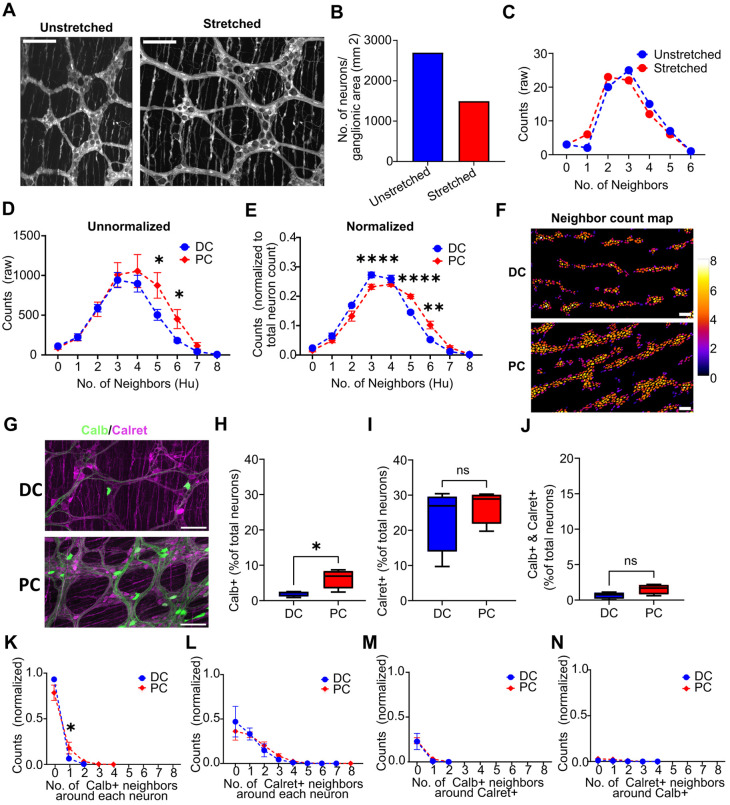
**Spatial analysis in GAT can adjust for non-uniform tissue stretching and can objectively describe region-specific differences in neuronal distribution.** (A) Both images show the same field of view of a myenteric plexus wholemount preparation of the Wnt1-cre:Rosa26-tdTomato mouse colon. The image on the left shows the specimen that was pinned in a petri dish (‘unstretched’), and the image on the right shows the same specimen in the presence of stretch. Scale bars: 100 µm. (B) Quantification of images as in A demonstrates that stretching the specimen leads to altered calculations of neuronal density (2696 neurons/mm^2^ versus 1493 neurons/mm^2^; *N*=1). (C) Proximal neighbor analysis shows similar distribution results for non-stretched versus stretched preparations (*N*=1). (D) Proximal neighbor distribution for the mouse proximal colon (PC) versus distal colon (DC) from a publicly available dataset (Hamnett et al., 2022a,b) without normalization to the total cell count shows that the PC has larger neuronal clusters, with a significantly large number of neurons with neighbor counts of 5 and 6 (*P=0.012 and *P=0.018, respectively; two-tailed unpaired *t*-test with Welch's correction). Mean±s.d.; *n*=4. (E) Normalizing the data in D for total neuron count (Hu-positive neurons) reveals that the DC has smaller neuronal clusters, with significantly more neurons having a neighbor count of 3, whereas the PC has larger neuronal clusters, with a significantly larger proportion of neurons having neighbor counts of 5 and 6 (****P=0.0007, ****P=0.00003 and **P=0.002, respectively; two-tailed unpaired *t*-test with Welch's correction). Mean±s.d.; *n*=4. (F) The difference in number of neighbors across regions can be visualized in a neighbor count map, where there are more orange- and red-colored neurons in PC (bottom) than in DC (top). Scale bars: 100 µm. (G) Representative images showing labeling of Calb and Calret in the mouse DC and PC. Scale bars: 100 µm. (H–N) Quantification and proximal neighbor analysis of Calb- and Calret-positive neurons in the mouse DC and PC. (H) Calb-positive neurons are a greater proportion of the total neurons in the PC compared to the DC (*P=0.046, two-tailed unpaired *t*-test with Welch's correction), whereas there is no significant difference in the distribution of (I) Calret-positive neurons or (J) Calret and Calb double-positive neurons (*P*=0.54 and *P*=0.08, respectively; two-tailed unpaired *t*-test with Welch's correction; ns, not significant). (K) This is reflected in the normalized PN distribution plots, where a higher proportion of neurons have one Calb-positive neighbor in the PC relative to the DC (*P=0.03; two-tailed unpaired *t*-test with Welch's correction). No regional differences in (L) Calret-positive neurons or (M,N) preferential association between Calret-positive and Calb-positive neurons are detected. Boxplots in H–J show the median (line), interquartile range (box) and minimum to maximum values (whiskers) of *n*=4. Data in K–N are presented as mean±s.d. of *n*=4.

To evaluate the robustness of spatial analysis with uneven tissue stretch, an image of the same region of a myenteric wholemount preparation of Wnt1-cre:Rosa26-tdTomato mouse colon was acquired under unstretched and stretched conditions. The neurons were manually counted by identifying cells with relatively low background intensities and large sizes (Fig. 3A). Stretch led to ∼55.3% increase in the tissue area examined (152,318 µm^2^ unstretched versus 245,652 µm^2^ stretched), total ganglionic area (26,573 µm^2^ unstretched versus 44,844 µm^2^ stretched) and cell densities in the ganglionic area (2696 neurons/mm^2^ unstretched versus 1493 neurons/mm^2^ stretched; Fig. 3B). Using the PN analysis, no difference was found between the average PNs around each neuron for stretched and unstretched tissue (2.76±1.25 stretched versus 2.98±1.21 unstretched; mean±s.d.; paired two-tailed Student's *t*-test, *P*=0.21; *N*=1). Importantly, the distribution of PNs for each cell was similar for both stretched and unstretched tissue (Fig. 3C), showing that GAT can capture the underlying cellular distribution even with significant differences in tissue area.

Spatial analysis in GAT enables the quantification of cellular distribution and can be used to evaluate differences across gut regions or in different disease states. To illustrate this objectively, images of the proximal colon (PC) and distal colon (DC) from a published dataset were analyzed (Hamnett et al., 2022a,b). Others have demonstrated that the PC has larger ganglia and greater neuronal density in comparison to the DC (Li et al., 2019; Nestor-Kalinoski et al., 2022). This was evaluated using GAT by quantifying the number of ganglia and the number of neurons per ganglion. There was a significantly larger number of ganglia in the DC compared to that in the PC (160.7±14.5 for DC versus 77.5±21.6 for PC; mean±s.d.; two-tailed unpaired *t*-test with Welch's correction, *P*=0.0008; *n*=4). However, the neuronal density per ganglion was not different when comparing the means (20.7±30.3 for DC versus 56.5±202.3 for PC; mean±s.d.; two-tailed unpaired *t*-test with Welch's correction, *P*=0.055; *n*=4). The neuronal count per ganglion showed considerable variability, likely due to the difference in ganglion size across each region, as reflected by the high s.d. values. To account for this, the median values were calculated. This revealed that the PC had a significantly lower number of neurons per ganglion, compared to that in the DC (8.3±1.2 for DC versus 3.6±1.9 for PC; median±s.d.; two-tailed unpaired *t*-test with Welch's correction, *P*=0.009; *n*=4). Neurons in the PC had greater number of neighbors than those in the DC (Fig. 3D) (raw counts of 503.8±68.6 versus 874.8±161 for five neighbors and 180.3±20.4 versus 451.5±120.2 for six neighbors for DC versus PC; mean±s.d.; two-tailed unpaired *t*-test with Welch's correction, *P*=0.012 for five neighbors and *P*=0.018 for six neighbors; *n*=4). This supports the observation of larger neuronal counts per ganglion in the DC. However, to account for differences in neuronal numbers in the PC and DC, the raw neighbor counts were normalized to total neuron count for each region. This revealed significant regional differences and a shift in the neuronal distribution (Fig. 3E). In the DC, a significant proportion of neurons had fewer neighbors, which is indicative of smaller ganglion size and, consequently, smaller neuronal clusters. The larger ganglion size and neuronal clusters were evident from the larger proportion of cells having more neighbors (Fig. 3E,F) in the PC relative to the DC (normalized counts of 0.27±0.01 versus 0.23±0.01 for three neighbors, 0.14±0.005 versus 0.2±0.007 for five neighbors and 0.05±0.004 versus 0.1±0.013 for six neighbors for DC versus PC; mean±s.d.; two-tailed unpaired *t*-test with Welch's correction, *P*=0.0007 for three neighbors, *P*=0.00003 for five neighbors and *P*=0.002 for six neighbors; *n*=4). Thus, the number of PNs is proportional to the cell density and size of the ganglion.

Region-specific differences in the distribution of neuronal subtypes could be reflective of the specific functions of the GI subregions (Hamnett et al., 2022b; Li et al., 2019; Nestor-Kalinoski et al., 2022). The regional distribution of the Ca^2+^-binding proteins Calb and Calret in neurons of the PC and DC was examined using images from the Hamnett et al. (2022a) dataset. The aim was to test the capacity of GAT to detect established regional differences in ENS distribution and investigate the relative distribution of these neuronal markers as they were co-labeled in the same tissue (Fig. 3G). Using GAT analysis, a higher proportion of Calb-positive (Calb+) neurons was detected in the PC compared to the DC (Fig. 3H; 1.99±0.78 for DC versus 6.25±2.71 for PC; mean±s.d.; two-tailed unpaired *t*-test with Welch's correction, *P*=0.046; *n*=4). Conversely, no significant differences in the number of Calret-positive (Calret+) neurons were detected across regions (Fig. 3I; 26.96±4.9 for DC versus 23.51±9.3 for PC; mean±s.d.; two-tailed unpaired *t*-test with Welch's correction, *P*=0.54; *n*=4), consistent with Hamnett et al. (2022a,b). In this dataset, preparations were co-labeled for Calb and Calret, enabling the use of GAT to assess neurons that were positive for both these markers. A very small proportion of Calret and Calb double-positive neurons were detected in PC and DC, and no significant difference was found in the distribution across regions (Fig. 3J; 0.65±0.46 for DC versus 1.54±0.71 for PC; mean±s.d.; two-tailed unpaired *t*-test with Welch's correction, *P*=0.08; *n*=4).

The distribution of Calb+ and Calret+ neurons was further assessed using spatial analysis in GAT. This revealed significantly greater numbers of neurons with one Calb+ neighbor in the PC compared to that in the DC (Fig. 3K; 0.18±0.06 for PC versus 0.06±0.02 for DC; mean±s.d.; two-tailed Mann–Whitney *U*-test, *P*=0.03; *n*=4). This finding aligns with the observation that the PC contains a greater proportion of Calb+ neurons (Fig. 3H), resulting in a larger number of Calb+ neighbors surrounding each neuron compared to the DC. No difference between regions was detected for neurons with Calret+ neighbors (Fig. 3L), which aligns with the finding of no difference in the proportion of Calret+ neurons between DC and PC (Fig. 3I). The spatial distribution of neurons that coexpressed Calb and Calret was also determined, with no significant difference between regions (Fig. 3M,N). Spatial analysis of Calret+ neurons relative to Calb+ neurons and vice versa did not reveal any regional differences, suggesting that Calret+ neurons do not preferentially associate with Calb+ neurons in either the PC or DC. These analyses demonstrate that spatial analysis using GAT effectively detects regional differences in neuronal and neuronal subtype distribution.

## DISCUSSION

GAT is a user-friendly Fiji-based software for studying the distribution of enteric neurons and their neurochemical coding in wholemount preparations of GI tissue in 2D. It uses DL models for segmentation of neurons and ganglia, which enables higher throughput and faster data extraction, making it possible to analyze large tissue areas, increasing the accuracy of neuronal density estimates (Fig. S1). Moreover, spatial analysis in GAT provides an objective means for characterizing the distribution of cells and distinct subpopulations. The DL models are coupled with a user-friendly workflow, thus enabling researchers with minimal computational expertise to adopt GAT for rapid and reproducible image analysis.

The availability of state-of-the-art user-friendly tools such as StarDist (Schmidt et al., 2018) and deepImageJ (Gómez-de-Mariscal et al., 2021), in combination with ZeroCostDL4Mic (von Chamier et al., 2021), was crucial for the development of GAT. The GAT enteric neuron models can be used within any software that supports StarDist, thus giving the user flexibility to generate custom analysis pipelines should they be required. The GAT software repository has models and scripts that are compatible with QuPath, a popular image analysis software for analyzing whole-slide images and large 2D images. Cellpose was used to compare the segmentation abilities of the DL models as it works readily on diverse datasets and the user interface makes it simple to use. It is unclear whether the Cellpose cyto2 model was trained on enteric neuronal datasets, which might explain the slightly lower performance metrics when compared to GAT (Fig. 2). In Cellpose v2.0, additional models trained on fluorescence images are available, which might show an improvement over cyto2 (Pachitariu and Stringer, 2022). Moreover, the best performance can be achieved by fine-tuning the Cellpose models, where the GAT training dataset can be combined with user-specific data to generate custom models (Pachitariu and Stringer, 2022). This could increase the accuracy of segmentation. However, performing quality checks with objective metrics is essential to evaluate the performance of DL models, as shown in Fig. 2A,C and Fig. S2.

StarDist was used for segmenting cells within GAT, as the software is optimized to detect objects with star-convex shapes such as cell nuclei. Thus, it is suitable for detecting enteric neurons, which have a circular shape (Schmidt et al., 2018). Other important benefits include the availability as a Fiji plugin; the ability to use StarDist within macros and/or scripts; the ability to tune the cell detection by changing the ‘probability’ value, allowing segmentation of images with varying labeling intensities or with high background noise; and the ability to detect overlapping cells. The overlap detection can be adjusted by changing the ‘overlap threshold’ value. This is particularly useful for analyzing tissue that has not been stretched appropriately or tissue from larger animals where the ganglia are thicker, resulting in greater overlap between cells in 2D. The caveat of using StarDist is that it can only be used for round cells and not for cells with complex morphology. This currently limits its use to enteric neurons, and other cell types where a nuclear stain is available, such as Sox10 for enteric glia. Thus, other cells with non-circular complex shapes, such as tissue resident macrophages or interstitial cells of Cajal, cannot be analyzed using the current pipeline. Future versions of GAT aim to add support for analyzing diverse cell types within the gut wall.

A limitation of using DL-based models in GAT is that they might not work across image types that GAT has not previously encountered. This variation could be images of wholemount preparations from other species (Fig. S2J), different regions or layers of the GI tract, or even images acquired using modalities that were not used for training GAT. This could be rectified by retraining the models with new data, but it might not always be feasible as this process is laborious and requires computational expertise. Given the evolving landscape of image analysis and cell segmentation software, GAT offers an option of importing custom segmentations for cells and ganglia directly into the analysis pipeline. This feature allows flexibility for the user to choose their preferred cell segmentation tool. As an example, this approach was used in Fig. 3. The neuron subtype model successfully segmented Calb+ neurons but was not consistent for Calret+ neurons, as the labeling was heterogenous. Similarly, segmentation of ganglia was not consistent using the ganglia model. To rectify this, QuPath was used for training an object classifier for Calret+ neurons and a pixel classifier for ganglia, thus enabling segmentation of Calret+ neurons and ganglia, respectively. The respective detections and annotations were exported from QuPath and reimported back into GAT during analysis.

One limitation of GAT is that the analysis workflow is currently designed for 2D images, as the Fiji StarDist plugin (v0.3.0) is limited to 2D datasets. Currently, GAT does not support importing three-dimensional (3D) segmentation from other software. When cells that occupy a 3D space are projected as a 2D image, they can often superimpose or overlap with each other, leading to challenges in accurately delineating and segmenting these cells (Fig. S2K). Separation of cells is more readily achieved with 3D data; however, only the Python implementation of StarDist supports 3D segmentation. Furthermore, cell shape and size are more accurately measured in 3D compared to 2D. One reason is that the volume measurements are less impacted by differences in tissue stretch compared to area measurements in 2D. 3D segmentation requires DL-based approaches that use high-quality 3D annotations, which is a time-consuming and laborious process. Several tools are available for annotating data in 3D (Berg et al., 2019; Boergens et al., 2017; Borland et al., 2021; Fedorov et al., 2012; Tasnadi et al., 2020). However, the success of DL models relies on the availability of large amounts of high-quality training data that account for the diversity of the markers used, the animal species studied, how the tissue was prepared and the variability in the instruments used for acquisition. Existing tools such as Cellpose (Stringer et al., 2021), 3D ImageJ suite (Ollion et al., 2013), and CLIJ or pyclesperanto (Haase et al., 2020a preprint, 2020b; https://github.com/clEsperanto/pyclesperanto_prototype) can be used for processing 3D data and enabling curation of labeled data. Commercial software such as Imaris has also been used to generate 3D masks for human enteric neurons (Parker et al., 2023). To enable ENS-specific analytical solutions, it is essential to have robust training data made accessible to the wider research community. For example, the GAT training dataset was deposited on Zenodo, an open data repository (see Materials and Methods). This has been utilized to generate custom cell segmentation models for image analysis software to quantify Ca^2+^ signaling in the gut (Barth et al., 2023). Moreover, the GAT models can be fine-tuned using custom data.

Manual analytical approaches to assess changes in the ENS are limited to cellular density and the number of neuron subtypes within the tissue. However, incorporating spatial analysis can reveal insights into cellular distribution, interactions and potential implications for function at a tissue level (Nestor-Kalinoski et al., 2022). Existing spatial analysis software solutions require significant computational expertise and may require optimization to study the ENS (Feng et al., 2023; Rose et al., 2020; Stoltzfus et al., 2020). Factors such as operator expertise, animal species being studied and the pathology investigated can affect how the tissue is stretched and prepared as a wholemount (Gomez-Frittelli et al., 2023; Kapur, 2013). This, in turn, can affect analysis and interpretation of cellular distribution (Fig. 3A–C). GAT accounts for possible variations in tissue stretch by using an enteric neuron-specific nearest neighbor distance threshold value. Differences in neuronal distribution were demonstrated objectively using the PN analysis in GAT. The PC was found to have relatively larger neuronal clusters compared to the DC, indicative of the large and small ganglion sizes in these regions, respectively. Despite the large variability in ganglion sizes within each region, the PN analysis effectively detected region-specific differences and proved to be a more robust measure than neuronal density measurements. Altered distribution of Calret+ and Calb+ neurons across the PC and DC were reflected in the spatial analysis, and no spatial association was found between neuronal subtypes. This approach can be extended to assess changes in the structure or morphology of the ENS across gut regions or in diseases including inflammatory bowel disease (Brierley and Linden, 2014), diabetes (Chandrasekharan et al., 2011; Demedts et al., 2013) and enteric neuropathies, such as Hirschsprung disease (Heuckeroth, 2018). For example, this analysis could be used to determine whether there are subtle differences, such as fewer nNOS-positive neuronal neighbors, which would be indicative of lower nNOS-positive neurons in these conditions. The parameters, such as the average PN distance, might need to be modified for human tissue, and this is configurable within GAT. Due to the analysis capabilities of GAT being limited to enteric neurons, the spatial analysis does not capture the complexity of cellular distribution and relationships with other cell types, such as enteric glia, macrophages and interstitial cells of Cajal. Incorporating additional spatial analysis metrics such as those related to cell colocalization or spatial heterogeneity (Feng et al., 2023), or the use of a spatial neighbors graph to understand neighborhood enrichment (Palla et al., 2022), could enable a more comprehensive and unbiased examination of cellular interactions and distributions in the gut.

GAT excels at segmenting neurons and works across images of varying staining qualities (Fig. S2H). It offers a faster alternative to manual analysis and has been designed with ENS-specific analysis solutions, such as studying neurochemical coding and spatial analysis of neuronal distribution. New features are regularly being introduced based on user feedback and experience. To enable compatibility for further analysis in other software, segmentation maps, cell outlines, cell type information and cell coordinates are extracted for each experiment during analysis. GAT is written in Fiji using the macro language, limiting the scope of the user interface and its interactivity. Future versions of GAT could use scripting languages in Fiji, which would offer greater flexibility in developing highly interactive user interfaces. Moreover, the availability of StarDist models enables the development of interactive workflows in napari and QuPath. GAT aims to create a toolbox that automates common image analysis tasks in ENS research, eliminating the burden of manual analysis. This allows scientists to spend more time interpreting biology and advancing scientific research.

## MATERIALS AND METHODS

### Datasets

The following previously reported datasets were used for training and benchmarking the neuronal, neuronal subset and ganglia segmentation models. (1) Lab 1: mouse (DiCello et al., 2020) , rat (Furness et al., 2023). (2) Lab 2: mouse (McQuade et al.. 2021). (3) Lab 3: mouse (Poon et al., 2022). (4) Lab4: human (Chen et al., 2023).

External datasets were also used for creating DL models. Data from the Stimulating Peripheral Activity to Relieve Conditions (SPARC) program website (https://sparc.science) were used for training the neuronal, neuronal subset and ganglia segmentation models. Within the SPARC portal, data from mouse (Nestor-Kalinoski et al., 2022; Kalinoski et al., 2021; Wang et al., 2021) and human myenteric plexus wholemount samples (Graham et al., 2020b) were used for curating training datasets.

The following previously reported datasets were used for proximal neighbor analysis in Fig. 3D–N: Calret+ and Calb+ images of DC and PC from dataset named EXP 174 in Hamnett et al. (2022a,b).

### Mice

Details of housing conditions and ethics statements for the previously reported mouse data used within this study can be found in the respective studies (DiCello et al., 2020; Hamnett et al., 2022b; McQuade et al., 2021; Nestor-Kalinoski et al., 2022; Poon et al., 2022).

For the data in Fig. 3A–C, B6;129S6-*Gt(ROSA)26Sor^tm9(CAG-tdTomato)Hze^*/J (Jackson Laboratory, Bar Harbor, ME; stock no. 007905) mice were crossbred with B6.Cg-*E2f1^Tg(Wnt1-cre)2Sor^*/J (Jackson Laboratory; stock no. 022501) mice. The F1 offspring expressed tdTomato within all ENS cells. The animals were handled in accordance with the institutional guidelines of the University of Tübingen, which conform to international guidelines. Mice were housed in standard plastic cages with standard bedding under a 12 h light-to-dark cycle at 22±2°C, 60%±5% humidity, with free access to food and water.

### Rat

The details of housing conditions and ethics statements for the previously reported rat data used within this study can be found in Furness et al. (2023).

### Human tissue

Pediatric colon tissue (used for model training data, Lab 1) was obtained from a 4-month-old male patient with rectosigmoid disease, with prior written informed consent from a parent/guardian [Royal Children's Hospital and Monash University (HREC: 38262 and 21091)], in accordance with principles expressed in the Declaration of Helsinki. The ethics statement for the previously reported data from adult human tissue can be found in Chen et al. (2023).

### Wholemount tissue preparation and immunohistochemistry

The antibodies used are listed in Table S4.

#### Mouse

The protocols used for dissection and immunohistochemistry of mouse myenteric wholemounts varied across labs and are summarized in the respective publications from each source (DiCello et al., 2020; Hamnett et al., 2022b; McQuade et al., 2021; Nestor-Kalinoski et al., 2022; Poon et al., 2022). For experiments using the Wnt1-cre:Rosa26-tdTomato mouse, the colon was excised from the abdomen of the mouse and incubated in preparation medium (HBSS without Ca^2+^or Mg^2+^, containing 1 µM nifedipine). The tissue was cleared of contents, cut open longitudinally and laid flat on a silicone elastomer-lined dish (Sylgard, Dow Corning, Midland, MI, USA) in preparation medium. To acquire images of unstretched colon, the tissue was laid flat without pinning, and images acquired using a Zeiss Axio Imager Z1 (20× HC PL APO NA 0.8) across various locations of the preparation. After image acquisition, the tissue was maximally stretched along the longitudinal and circumferential axis and pinned mucosa downwards on the silicone elastomer-lined dish. The stretched preparation was fixed in 4% paraformaldehyde for 10 min at room temperature, and subsequently rinsed [3×1 h in phosphate-buffered saline (PBS)]. Images of the stretched preparation were acquired by manually locating the corresponding region from the unstretched preparation.

#### Human

The protocol for used for adult human colon samples can be found in Chen et al. (2023). For the pediatric human colon tissue, surgical specimens were transferred into PBS (pH 7.2) containing nicardipine (10 µM). The tissue was opened to flat sheets along the longitudinal axis, maximally stretched along the longitudinal and circumferential axis, and pinned mucosa downwards on a silicone elastomer-lined dish (Sylgard, Dow Corning, Midland, MI, USA). The tissue was fixed in 4% paraformaldehyde overnight at 4°C, and subsequently cleared (3× 1 h in PBS). The mucosa, submucosa and circular muscle were removed by sharp dissection to prepare longitudinal muscle-myenteric plexus (LM-MP) wholemount preparations. The tissue preparations were blocked and permeabilized overnight in blocking buffer (PBS with 5% normal donkey serum, 0.1% w/v sodium azide and 0.5% Triton X-100). The samples were incubated in HuC/D primary antisera diluted in blocking buffer (5–7 days at 4°C; Table S4). The LM-MP preparations were washed (3× 30 min, PBS with 0.1% w/v sodium azide) and incubated in secondary antibodies (PBS with 0.1% w/v sodium azide, overnight at 4°C). They were then washed (PBS, 2× 30 min), followed by labeling with the nuclear marker 4',6-diamidino-2-phenylindole (DAPI; 1:500 in PBS, 1 h at room temperature). The samples were then washed once in PBS and mounted in buffered glycerol (nine parts glycerol and one part PBS; pH 8.5–9.0).

### Image acquisition

Datasets generated for this study were acquired using different instruments. This enables the DL models to generalize well and work across a variety of data acquired with commonly used microscopes.

Images of mouse tissue were acquired using a Leica TCS-SP8 confocal system (20× HC PL APO NA 1.33, 40× HC PL APO NA 1.3), a Leica TCS-SP8 Lightning confocal system (20× HC PL APO NA 0.88), a Zeiss Axio Imager M2 (20× HC PL APO NA 0.3) or a Zeiss Axio Imager Z1 (10× HC PL APO NA 0.45). Human tissue images were acquired using an Olympus IX71 microscope (10× HC PL APO NA 0.3) (Chen et al., 2023) or a Leica TCS-SP8 confocal system (20× HC PL APO NA 1.33, 40× HC PL APO NA 1.3). Acquisition of SPARC datasets used a Leica TCS SP5 laser scanning confocal microscope (20× NA 0.70, 40× NA 1.25 or 63× NA 1.4) (Nestor-Kalinoski et al., 2022) or a Zeiss LSM 710 confocal microscope (10× and 20× PL APO) with z-axis increments of 4 μm (10× objective) or 1 μm (20× objective) (Graham et al., 2020b).

### Software

#### Training data

Training data were generated using custom scripts written in Fiji macro language, provided with GAT (GAT→Tools→Data Curation). Briefly, the entire image or a portion of the image was selected for annotation. This was followed by manually outlining neurons using the drawing tools and saving them in the ROI Manager. For the enteric neuron datasets, the annotated images of varying sizes were saved as raw images and as segmented label images, where each neuron had an individual pixel value. In the label images all pixels with value 1 belong to neuron 1, all pixels with value 2 belong to neuron 2 and so on. The binary masks were saved for segmentation of ganglia. The annotation was performed and verified by at least two researchers experienced with the identification of enteric neurons and ganglia. Briefly, the outlines from the label image were overlaid on the raw images and verified for each cell using Fiji. The ‘Verify Images Masks’ macro within GAT→Tools→Data Curation was also used. For images that had DAPI labeling, the neuronal nuclei were used to delineate overlapping cells. Incorrect regions of interest (ROIs) were deleted and redrawn using the Oval or Freehand drawing tools in Fiji.

#### Enteric neuron models

The training images (Tables S1 and S2) were normalized to account for any variations in the sizes of cells in pixels due to image acquisition conditions, such as resolution and magnification, and species differences. The images from mouse and rat tissue were scaled to a pixel size of 0.568 µm per pixel, whereas the images from human tissue were scaled to 0.9 µm per pixel due to the larger cell sizes. This rescaling process ensured that the training images had a uniform average neuron area of 701.2±195.9 pixel^2^ (mean±s.d., 6267 cells) irrespective of image magnification or animal species. A similar approach was used to generate a training dataset for the enteric neuron subtype model. This training dataset contained images of neurons expressing various neurochemical markers, including Calb, nNOS, Calret, choline acetyltransferase (ChAT), delta-opioid receptor (DOR, also known as OPRD1), mu-opioid receptor (MOR, also known as OPRM1), neurofilament 200 (NF200, also known as NEFH) and somatostatin. The average cell area in the neuronal subtype dataset was 880.9±316 pixel^2^ (mean±s.d., 924 cells), with around 56.6% of the cells being nNOS positive. Thus, nNOS cells were overrepresented in the dataset. The StarDist v0.3.0 Fiji plugin was used for inference.

#### Ganglia model

The ganglia model was trained on images (Table S3) with both the pan-neuronal marker Hu and a second marker that labeled the neuronal or glial fibers. Regions where both markers were co-distributed were manually labeled as ganglia. Each ganglion was demarcated from a closely apposing ganglionic structure if an interganglionic strand separated them and/or if the gap was greater than the diameter of a single cell. The markers used for the identification of ganglionic structures consisted of any of the following: protein gene product 9.5 (PGP9.5, also known as UCHL1), nNOS, glial fibrillary acid protein (GFAP), S100b, Tuj1 or NF200. The deepImageJ v2.1.12 Fiji plugin was used for inference.

#### DL models and software

StarDist v0.7.3 (Schmidt et al., 2018) was used via ZeroCostDL4Mic v1.13 notebooks (von Chamier et al., 2021) within Google Colab for training the 2D segmentation models for enteric neurons and neuronal subsets. The ganglia model was trained in Google Colab using a 2D UNet architecture (Ronneberger et al., 2015) and exported to be readily used within deepImageJ (Gómez-de-Mariscal et al., 2021). The notebooks used for training the models, the training datasets used, training reports, model quality reports, and the models are deposited online at Zenodo (Sorensen et al., 2022; https://doi.org/10.5281/zenodo.10460434).

Cellpose (v0.7) was used as a baseline for comparing cell segmentation in this study. It is a generalist cell segmentation solution aimed at analyzing a wide variety of cell types (Stringer et al., 2021). It is not known whether Cellpose has been trained on enteric neuron images.

All training data and DL models are deposited at Zenodo (Sorensen et al., 2022; https://doi.org/10.5281/zenodo.10460434). Some of the essential training parameters are listed below. The training parameters used for the enteric neuron StarDist model were: number of epochs, 400; patch size, 240×240; batch size, 2; number of steps, 86; percentage validation, 10; n rays, 96; grid parameter, 2; initial learning rate, 5×10^−5^. The training parameters for the enteric neuron subtype StarDist model were: number of epochs, 300; patch size, 240×240; batch size, 1; number of steps, 171; percentage validation, 10; n rays, 96; grid parameter, 2; initial learning rate, 5×10^−5^. The training parameters for the ganglia UNet model were: number of epochs, 40; patch size, 768×768; batch size, 4; number of steps, 81; percentage validation, 0.1; initial learning rate, 0.0002; pooling steps, 0; min fraction, 0.

#### COUNTEN analysis

For benchmarking with COUNTEN (Kobayashi et al., 2021), the software was accessed from https://github.com/KLab-JHU/COUNTEN. The analysis used the default values of sigma and the minimum number of neurons per ganglion, set at 7 and 3, respectively. A Google Colab notebook was designed to enable interactive analysis with an option for batch analysis, which can be accessed from https://github.com/pr4deepr/COUNTEN.

#### Analysis of calbindin- and calretinin-positive cells

A publicly available dataset (Hamnett et al., 2022a) was used for analyzing Calb- and Calret-positive cells. Image files with Calb and Calret co-labeling (EXP 174) were analyzed using a combination of QuPath v0.4.3 (Bankhead et al., 2017) and GAT. Due to inconsistent segmentation of the ganglia using the ganglia model, a pixel classifier was trained in QuPath to identify ganglia based on the co-expression of Hu, Calb and Calret. The resulting annotations were exported from QuPath as Fiji-compatible ROIs, which were subsequently imported into GAT for analysis. Similarly, the enteric neuron subtype model detected Calb-positive neurons but did not reliably detect Calret-positive cells. To address this, an object classifier was trained in QuPath to detect Calret-positive neurons. The neurons were initially detected using the enteric neuron StarDist model based on the Hu channel, followed by the application of the object classifier. Only the Calret-positive ROIs were extracted from QuPath and imported into GAT for analysis.

The results for each replicate were merged into summary data using the scripts within GAT→Analysis. The summary data were analyzed in Python (v 3.9.15) and pandas (v 2.0.2) and visualized using seaborn (v 0.12.2). The analyzed data were exported, and statistical analysis was performed in GraphPad Prism (v 9.5.1).

#### Effects of varying magnification and sampling

An image of a myenteric wholemount of the mouse colon (13.9 mm^2^) labeled with Hu was used with QuPath v0.3.2 (Bankhead et al., 2017) to test the effects of varying magnification and sampling on estimated cell counts (Fig. S1B–E). A whole image annotation was created and then divided into tiles, where each tile had areas of 775,918 µm^2^, 338,116 µm^2^ and 150,274 µm^2^, thus simulating 10×, 20×, and 40× objective magnifications, respectively. Tiles at the edges of the tissue that were below 60,000 µm^2^ in area and areas with uneven staining were excluded in the subsequent calculations. To perform cell segmentation, a custom groovy script in combination with the StarDist 2D enteric neuron model converted into ONNX format was used in QuPath (https://github.com/pr4deepr/GutAnalysisToolbox/tree/main/QuPath_workflow). The parameters used for segmentation were a rescaling factor of 1 and a probability of 0.65. Once segmentation was performed, the cell numbers were saved using the measurement tables. Once cell counts were estimated, a custom Python script was used to choose random tiles and estimate mean values. The Python code and analysis are deposited at Zenodo (https://doi.org/10.5281/zenodo.13932357; version 1.0; Rajasekhar et al., 2024).

#### Evaluation of Cellpose and StarDist segmentation

The segmentation metrics for Cellpose cyto2 and StarDist enteric neuron model were further evaluated using Adapted Rand error, VOI merge and VOI split on the test data from the enteric neuron model training. Adapted Rand error assesses the overall segmentation quality, whereas VOI merge and VOI split assess errors associated with cell merging and splitting, respectively (Wolny et al., 2020). The evaluation script from plant-seg-tools GitHub repository (https://github.com/hci-unihd/plant-seg-tools/tree/main) was used to evaluate the segmentation results from the Cellpose cyto2 and enteric neuron StarDist model against the ground truth data (Fig. S2E–G).

#### Benchmarking on human data

Two publicly available datasets containing images of neurons in the human myenteric plexus were used to evaluate the GAT enteric neuron model, which contained widefield (Parker et al., 2022) and confocal image datasets (Chen et al., 2023) (Fig. S2J,K). Maximum-intensity projection images of the confocal datasets were generated in Fiji. Manual cell counts for widefield datasets were performed in Fiji, whereas the cell counts for the 3D datasets were obtained from their corresponding Imaris files (Parker et al., 2023). The rescaling factor was optimized for each dataset using the ‘Test neuron rescaling’ option within GAT. For neuronal counts in GAT, a rescaling factor of 0.5 was used for the widefield images, and the default of 1 was used for the confocal image datasets with a probability of 0.65.

#### Other

ChatGPT (GPT-3.5) was used for initial formatting and editing of the manuscript. The outputs have been edited, and the authors take full responsibility for the content of this publication.

The brightness of the microscopy images in Figs 2F and 3G was adjusted in a linear and uniform manner using the Brightness/Contrast dialog in Fiji to enable better contrast for visualizing the ganglia.

## Supplementary Material



10.1242/joces.261950_sup1 Supplementary information
