# Supplementary material for: Gut Analysis Toolbox – automating quantitative analysis of enteric neurons
Source: J Cell Sci. 2024 Oct 30;137(20):jcs261950. doi: 10.1242/jcs.261950 (PMC11698042; doi:10.1242/jcs.261950)
Supplement: Supplementary information [file joces-137-261950-s1.pdf]

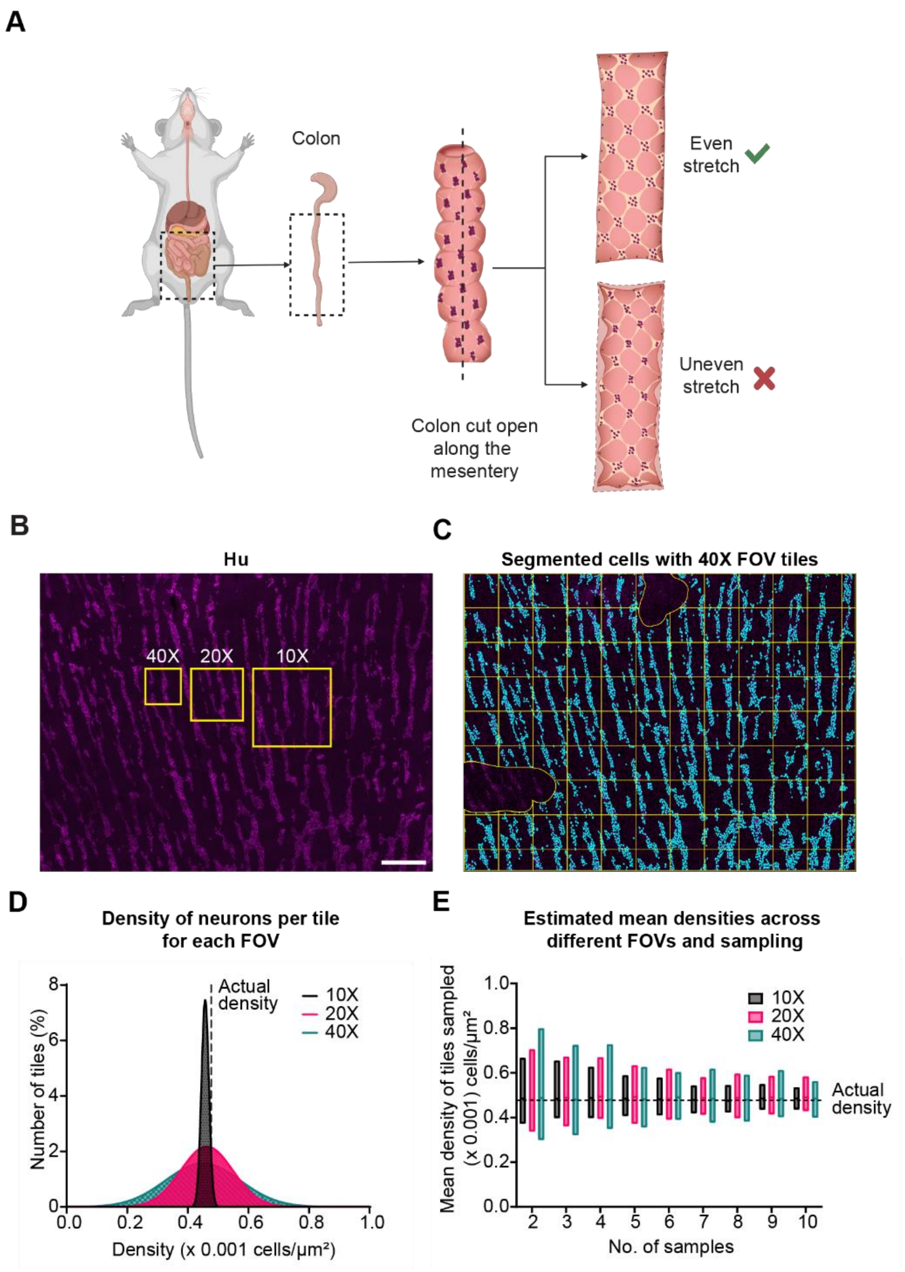

Fig. S1. Variability in ENS neuronal density estimates is influenced by sample preparation, tissue stretch and magnification levels. (A) For wholemount preparations, the isolated intestine is cut open along the mesenteric border, stretched, and pinned as a flat sheet. Individual expertise can determine the quality of this dissection and preparation (Created in BioRender. BioRender.com/y41w773) **(B)** An image of mouse colon tissue

Journal of Cell Science • Supplementary information

labeled for the pan-neuronal marker Hu (cell density 13.9 mm^2 area). Overlays represent the area occupied by different magnifications (10X, 20X, and 40X, scale bar = 100 µm). **(C)** Using a StarDist-trained model, QuPath was used to segment 6650 neurons (blue overlay). Regions with inconsistent staining or dissection were excluded (yellow overlay). Each yellow tile is representative of a 40X field of view (FOV) (**D)** For each individual tile, the density of neurons was calculated to demonstrate that 20X and 40X had a larger variation compared to 10X. **(E)** To illustrate the effect of sampling on averaged neuronal density estimates, images from each magnification were randomly sampled and mean cell densities were estimated across different sampling numbers (2-10 images). This was repeated 100 times for each FOV and sampling number. Each bar in the graph represents mean estimates of mean densities for each sampling and FOV choice. Bars represent the minimum and maximum value. The black dotted line indicates the cell density calculated from the entire field of view in **B**, 0.4768 (x 0.001 cells/μm^2). Thus, it is recommended to use larger FOVs, such as a 10X or 20X magnification, and increased tissue sampling to ensure accurate cell density estimates.

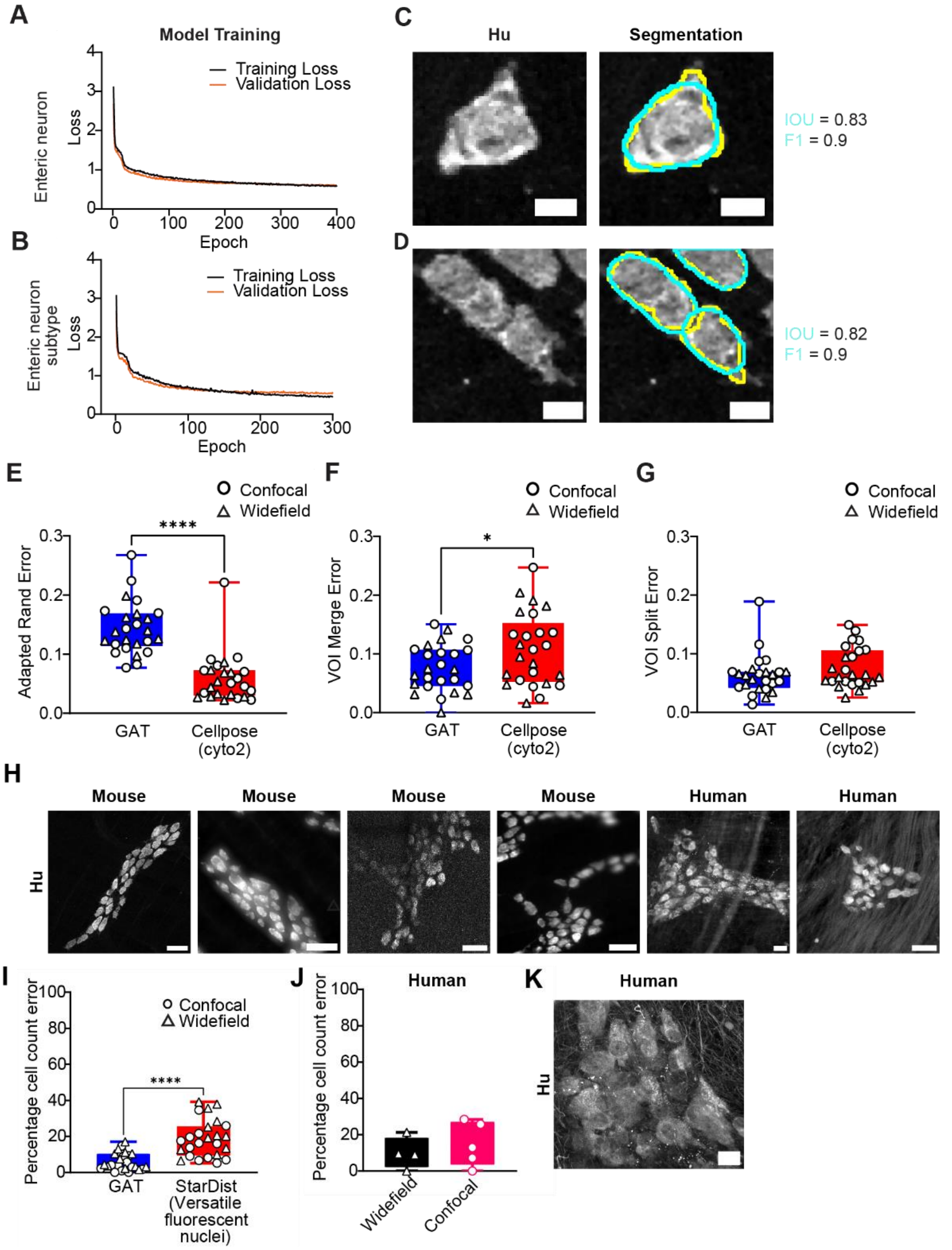

Fig. S2. Summary of StarDist enteric neuron model training and evaluation of the segmentation quality with representative Hu-labelled enteric neurons from the training dataset showing the varying staining qualities. Training loss of the StarDist enteric neuron model (**A**) and enteric neuron subtype model (**B**). (**C, D**) Comparison between representative StarDist segmentations (cyan outline/text) and manual annotation (yellow outline). The StarDist predictions have smoother boundaries but do not capture the original cell contours (Scale bar = 10 µm). (**E**) Cellpose had a lower Adapted Rand Error, indicating higher overall

Journal of Cell Science • Supplementary information

segmentation quality than the StarDist model (Mean ± s.d., $p=7.6x10^{-9}$, two tailed unpaired t-test with Welch's correction, n=25 images, 3830 neurons). However, this could be because Cellpose is better at predicting accurate cell borders compared to how StarDist approximates the cell shape using star convex polygons. The Cellpose model had larger merge and split errors (**F** & **G,** Mean ± s.d., p=0.17 and p=0.03 respectively, two tailed unpaired t-test with Welch's correction, n=25 images, 3830 neurons), which may explain the higher percentage of cell count error presented in **Fig. 2**. (**H**) Sample images of enteric neurons from the myenteric plexus of the mouse and human colon with varying qualities of Hu immunostaining from the enteric neuron model training dataset (50 µm scale bar; AutoScale in Fiji was used to enhance the contrast). **(I)** Comparison of percentage cell count error for GAT Stardist enteric neuron model vs the default versatile fluorescent nuclei model (Mean ± s.d., p=0.000015, two tailed unpaired t-test, n=25 images, 3830 neurons). (**J**) The performance of GAT enteric neuron model evaluated on images of myenteric wholemounts from the human colon acquired using widefield (4 images, 313 neurons) and confocal microscopes (5 images, 228 neurons). The large variations in percentage cell count error observed can be explained by the 3D distribution of neurons in the human ganglia. (**K**) A representative image of a myenteric wholemount from a human colon shows the heavy overlap between enteric neurons in a maximum projection image. This overlap is due to the 3D organization of the cells in the ganglia (Scale bar = 20 µm).

Journal of Cell Science • Supplementary information

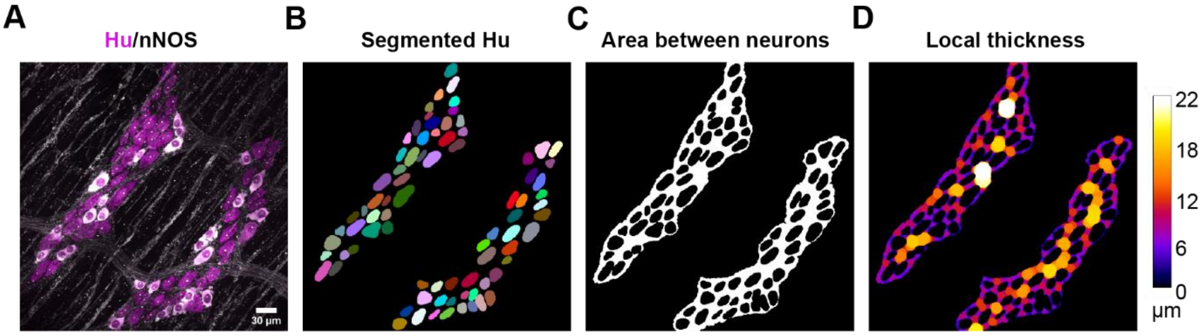

Fig. S3. Average proximal neighbor distances can be determined by calculating the local thickness within the ganglia. The distances between neurons in a ganglion can be determined by segmenting the neurons and the ganglia (**A, B**) and measuring the ganglionic area, excluding neuronal soma **(C)**. **(D)** The local thickness map can be used to derive the interneuronal distances. This was calculated by applying the "Local Thickness" plugin (Dougherty and Kunzelmann, 2007) on the binary image **(C)**. The "Local Thickness (complete process)" option in Fiji was used to compute the local thickness map as shown in **D** (Scale bar = 30 µm).

Table S1. Summary of the images used for training the enteric neuron StarDist model.

Species	Lab 1	Lab 2	Lab 3	SPARC	Lab 4	Total images
**Mouse**	44	15	6	1	-	**66**
**Rat**	2	-	-	-	-	**2**
**Human**	2	-	-	6	8	**16**
**Magnification**	40X/20X	10X	20X	20X/40X	10X	-
**Modality**	Confocal	Widefield	Widefield	Confocal	Widefield	-
**Total images**	**48**	**15**	**6**	**8**	**7**	**84**

All images contain cells labeled with the pan-neuronal maker Hu. The number of images are mentioned at the bottom of the columns for Species and Total images. The microscope configuration is listed in the methods section under Image acquisition.

Table S2. Number of images used for training the enteric neuron subtype StarDist model.

Data source	nNOS	MOR	DOR	ChAT	CalR	CalR	NFM	CGRP	SST	Total/ source
**Lab 1**	4	2	1	2	1	-	-	-	-	10
**Lab 2**	16	-	-	-	-	-	-	-	-	16
**Lab 3**	3	-	-	-	5	9	6	-	-	23
**Lab 4**	-	-	-	-	-	-	-	-	-	0
**SPARC**	1	-	-	3	-	1	-	1	1	7
**Total/ marker**	24	2	1	5	6	10	6	1	1	**56**

The microscope configuration is listed in the methods section under Image acquisition.

Journal of Cell Science • Supplementary information

Table S3. Number of images used for training the ganglia model

Data source	PGP9.5	GFAP	Wnt1Cre-GFP	ChAT	NF200	5HT	CGRP	NPY	nNOS	s100b	Tuj1	Total / source
**Lab 1**	12	28	3	-	-	-	-	-	5	-	-	48
**Lab 2**	-	-	-	-	-	-	-	-	12	-	-	12
**Lab 3**	-	-	-	-	-	-	-	-	-	-	-	0
**Lab 4**	-	-	-	1	1	2	1	1	-	-	-	6
**SPARC**	-	-	-	4	-	-	-	-	-	3	3	10
**Total/ marker**	12	28	3	5	1	2	1	1	17	3	3	**76**

Each marker was used in combination with Hu to generate training data for the 2D U-Net ganglia model implemented using deepImageJ. The microscope configuration is listed in the methods section under Image acquisition.

Journal of Cell Science • Supplementary information

Table S4. Primary antibodies used for immunofluorescent labeling of wholemount preparations.

Target	Host Species	Dilution	Supplier	Clone/Catalog number	RRID/ Reference
HuC/D	Mouse	1/200	Thermo Fisher	A-21271	AB_221448
HuC/D	Human	1:5000	Gift from Vanda Lennon, Mayo Clinic	N/A	AB_2314657
nNOS	Sheep	1:1000	Emson	K205	AB_2895154
Calbindin	Rabbit	1:1600	Swant	CB-38a	AB_10000340
NFM	Rabbit	1:500	Merck	AB1987	AB_91201
Calretinin	Goat	1:1000	Swant	CG1	AB_10000342
nNOS	Sheep	1:1000	Gift from Dr Piers Emson		AB_2314960

The secondary antibodies used were donkey anti-human 594 (Jackson ImmunoResearch), donkey anti-sheep 647 and donkey anti-sheep 488 (Thermo Fisher Scientific). Streptavidin-AMCA was used with donkey anti-mouse biotin for Hu in the adult human colon preparations (Chen et al., 2023).

Journal of Cell Science • Supplementary information
