# Supplementary material for: Gut Analysis Toolbox – automating quantitative analysis of enteric neurons
Source: J Cell Sci. 2024 Oct 30;137(20):jcs261950. doi: 10.1242/jcs.261950 (PMC11698042; doi:10.1242/jcs.261950)
Supplement: Supplementary file 1 [file joces-137-261950_review_history.pdf]

Gut Analysis Toolbox - automating quantitative analysis of enteric neurons

Luke Sorensen, Adam Humenick, Sabrina S. B. Poon, Myat Noe Han, Narges S. Mahdavian, Matthew C. Rowe, Ryan Hamnett, Estibaliz Gómez-de-Mariscal, Peter H. Neckel, Ayame Saito, Keith Mutunduwe, Christie Glennan, Robert Haase, Rachel M. McQuade, Jaime P. P. Foong, Simon J. H. Brookes, Julia A. Kaltschmidt, Arrate Muñoz-Barrutia, Sebastian K. King, Nicholas A. Veldhuis, Simona E. Carbone, Daniel P. Poole and Pradeep Rajasekhar
DOI: 10.1242/jcs.261950

Editor: Guillaume Jacquemet

Review timeline

Original submission:	18 January 2024
Editorial decision:	4 March 2024
First revision received:	29 July 2024
Accepted:	27 August 2024

Original submission

First decision letter

MS ID#: JOCES/2024/261950

MS TITLE: Gut Analysis Toolbox: Automating quantitative analysis of enteric neurons

AUTHORS: Luke Sorensen, Adam Humenick, Sabrina S.B. Poon, Myat Noe Han, Narges Sadat Mahdavian, Ryan Hamnett, Estibaliz Gómez-de-Mariscal, Peter H Neckel, Ayame Saito, Keith Mutunduwe, Christie Glennan, Robert Haase, Rachel M. McQuade, Jaime P.P. Foong, Simon J.H. Brookes, Julia A. Kaltschmidt, Arrate Muñz-Barrutia, Sebastian K. King, Nicholas A. Veldhuis, Simona E. Carbone, Daniel P. Poole, and Pradeep Rajasekhar
ARTICLE TYPE: Tools and Resources

We have now reached a decision on the above manuscript.

To see the reviewers' reports and a copy of this decision letter, please go to: https://submit-jcs.biologists.org and click on the 'Manuscripts with Decisions' queue in the Author Area. (Corresponding author only has access to reviews.)

As you will see, the reviewers gave favourable reports but raised some critical points that will require amendments to your manuscript. I hope that you will be able to carry these out because I would like to be able to accept your paper, depending on further comments from reviewers.

Please ensure that you clearly highlight all changes made in the revised manuscript. Please avoid using 'Tracked changes' in Word files as these are lost in PDF conversion.

I should be grateful if you would also provide a point-by-point response detailing how you have dealt with the points raised by the reviewers in the 'Response to Reviewers' box. Please attend to all of the reviewers' comments. If you do not agree with any of their criticisms or suggestions please explain clearly why this is so.

© 2024. Published by The Company of Biologists under the terms of the Creative Commons Attribution License (https://creativecommons.org/licenses/by/4.0/).

Reviewer 1

Advance summary and potential significance to field

Sorensen and colleagues have presented a new tool in the form an ImageJ/Fiji plugin to assist in the segmentation and analysis of enteric neurons within the Gut. The underlying premise of this tool called GAT (Gut Analysis Toolbox) is that it speeds up the processing time required to detect, count and measure neurons within light microscopy micrographs, in a relatively straightforward way. Improvements in using pre-trained models to identify cells and objects within images have exploded in recent years, with more recent improves allowing 3rd parties to train their own models on new cell types beyond those originally conceived. Often pre-training a new model, or even implementing this model into a plugin/tool is not easy, which is where GAT steps in. Building on existing published and widely used tools such as StarDist, the Sorensen et al present a toolbox or suite of small scripts to aid the user in tuning a pretrained model to identify and quantify neuron cells within a ganglia. Any tool that successfully lowers the barrier of entry to end users and helps to eliminate user bias in quantification is a significant step in the right direction for the fields of image analysis and neuroscience and is therefore a suitable publication to this journal.

Comments for the author

The GAT (Gut Analysis Toolbox) presented here by Sorensen et al is of value to the community and is suitable to JCS, however a number of revisions must be addressed before I would recommend it for publication. The article is well written and provides a strong case for why this tool is required, however when using the tool, a number of issues were encountered that I believe need to be fixed to ensure usage and adoption by non-expert users. The data presented is compelling and of acceptable standard for the journal, with clear and easy to follow figures. The inclusion of externally published datasets provides compelling use-case and quality of the tool provided.

My following comments are broken down into the manuscript, and the tool itself.

-Overall, the publication reads well with minor typos/revisions clarifications (set out below), and is well paced inducing the different possibilities of the GAT with enough information to get the hooked reader to visit the GitHub repository to download and begin exploring the tool.

-While the authors discuss two published methods for performing this kind of analysis (Cellpose & COUNTEN) comments or inclusion of other methods that have been developed for ENS image analysis such as Durand T, et al Communications Biology 2023 and/or calcium imaging of the ENS (Cavin, Jean-Baptiste et al The Journal of Physiology, 2023) should not be ignored. It would also be advantageous to compare or comment on commercial offerings beyond just Apeer such as Imaris/Arivis/Avia as used in the paper Herath Madushani, Biomolecules 2023 on finding/counting objects within regions (some of which also include Stardist or other deep learning models).

-A number of references don't list the available DOI

-Note that the acknowledgements, funding and author contribution sections are not provided.

Minor corrections

+line 108 - Sentence beginning "Multiple variables..." requires re-wording shortening, or more clarification.

+line 130 - link to GitHub repository, reference link to deepImageJ is broken

+line 649 - Table S1, it would be advantageous to include Numerical aperture (the determinant of resolution) and not just magnification information.

+line 185 - I would caution using the word 'significantly' when referring to a graph with I assume n=1 samples.

+Line 265 remove duplicate with

© 2024. Published by The Company of Biologists under the terms of the Creative Commons Attribution License (https://creativecommons.org/licenses/by/4.0/).

+Line 298 referencing panel 3A, is it not stretched or poorly stretched?

+Line395 celltypes to cell types

+Lines 517-528 more information on acquisition systems is required, software stand, immersions, detector gating & type, etc

+Line 540 typo inusing

+Line 584 typo An google?

+Line 650 ?mentioned under? should be changed to ?mentioned for? the species?

+Line 676 ?values.The? to ?values. The?

+Line679 (and others) examples of CellPose / Cellpose please be consistent, note most other published references use ?Cellpose?

+Line 758 please confirm if this is tested or untested.

+Line 764 please confirm if this is tested or untested.

+Line 768 please confirm if widefield were 2D single slices or 2D projections

+Line 782, a comment or data on the observed S:N for the different fluorophores & modalities would be useful to the reader here.

+Figure 2 significance symbols in panel C have not rendered correctly.

+Figure S1 B/C requires scale bars

For the GAT plugin itself, I believe requires the most work and revision before this publication could be accepted as I encountered a number of bugs, crashes and issues during testing that for a non-expert user would render this plugin inoperable.

-I initially had difficulty getting GAT to install properly within my existing daily-driver Fiji instance which has a number of existing plugins and tools installed, and instead had to get it working with a fresh install, this should be at least noted in the guide.

-The authors highlight that Stardist can be called from macros automatically and can be fed information from within the command, however their own tool doesn?t support that functionality. I fail to see the scenario where tools such as the Analyse Neurons, wouldn?t want to be batch scriptable or callable via the macro language. Such an extension I believe would be very valuable to the GAT plugin now at the time of publication.

-In numerous tools/scripts called from the GAT menu in Fiji, dialogs would popup (waitforuser commands) that are not clear. For example 'Check image to select the right channel' does not provide a clear indication what the right channel is? This should instead reference the relevant processing step such as Confirm which displayed channel has labelling for Ganglia Staining, Neuron Staining etc).

-If I select the option not to open an image by providing a filepath and instead check that the image is open, an additional checkbox to not close (or to close all) outputs would be nice. In your worked example video on the 2nd module of the documentation you show creating the output via GAT> Analyse Neurons then go on to use the output which was closed by the tool? Instead if I use the open image for the plugin tool (especially for parameter determination), it could be safely assumed that once that parameter is determined, I will execute the relevant tool and thus need the file open.

© 2024. Published by The Company of Biologists under the terms of the Creative Commons Attribution License (https://creativecommons.org/licenses/by/4.0/).

-For the tool 'Analyse Neurons (Multi-Channel).ijm' I have identified that it has some error catching requirements that need to be addressed. If I don?t select theCalculate_Neuron_Subtype checkbox or Enter_channel_details_now this will cause the tool to crash (even leaving NA in the text fields results with an error: 'Array expected in line 209,'. Upon investigating the code I don?t see any checks that NA is in the Textbox. I can check the boxes listed above and it runs fine asking for the probability sliders and follows expected behaviour. My quick assumption is to put lines 189/190 into the if conditional from line 173 or provide a default array if not being used, and provide a catch that if NA is registered to ignore it.

-The Test_Ganglia_Segmentation_Hu.ijm script can run with 'Image_already_open' but still requires a saved set of ROIs (and thus the script already being run).

Perhaps make this clearer (and in other menus) by having an '*'for required or similar (understanding that Fiji menu?s do have limitations).

+Also, if no file path is given, however image is open option is selected the script crashes as 'max_proj' isn?t defined.

-ROIs when saved as outputs are sometimes named (Ganglia; 'Label_' or Hu ROIs; '1') or not (re)named (nNOS ROIs; e.g. '0088-0573') it would be good if they were labelled more clearly. Also providing a multi-channel jpg with the respective ROIs displayed as an overlay would be useful for each individual ROI set, for example A flattened composite image with ganglia ROIs shown, an additional flattened composite image of Neuron ROIs shown etc.

-Having non-unique outputs (either files or directories) can cause issues with Fiji macros. For example, if I run the analysis, open the csv and find something isn't quite right, and I need to run the macro again but don?t close the .csv output file it will crash the macro. This occurs because excel or whichever program locks the file while its open, Fiji then tries to write to that file as per the commands in the script resulting in a crash. To overcome this, including date/time or time of day in milliseconds is a common way around this.

-When identifying ganglia within the image, often the selection would be quite noisy (and not correct) which would throw an error saying 'Max number of label reached (65535) in line 121' [or equivalent]. The error doesn't cause the macro to crash but may concern unfamiliar users. Perhaps a lower floor limit on ganglia size could be used to filter these issues?

Quality of Life improvements include:

-In the Help & Support menu, rather than printing results to a table that is not clickable/selectable, I would provide submenus with direct hyperlinks. This can be a number of python scripts calling the hyperlinks. E.g. import webbrowser webbrowser.open("https://gut-analysis-toolbox.gitbook.io/docs/")

Or invoking a dialog with HTML enabled if text descriptors are required

-The dialog for ?Enter channel number for Cell should auto-populate with the active channel number from the image, e.g. Image opens to CH1, in confirming the channel I change it to CH2, this default dialog value should be determined by the command Stack.getPosition(channel, slice, frame); which would be placed into the dialog as the default text.

-Provide a copy of the original ROIs and then any user-updated ROIs for transparency, for example a way of determining which were deleted by the user rather than just the text information stored in the log window.

Minor improvements/reviews

-On a few occasions using the varying tools for identifying the ganglia would select the whole image (instead of just the ganglia portion). Perhaps having a check / popup / flag to say ?It looks like the ganglia area matches the whole image area, that?s not very likely?? then allow for a different segmentation choice/confirmation.

© 2024. Published by The Company of Biologists under the terms of the Creative Commons Attribution License (https://creativecommons.org/licenses/by/4.0/).

-A text file with the provided example/test data should be provided to ensure non-expert users can understand which channel is which, for example filename.tif, ch1 = 'XYZ label' Ganglia, ch2 = 'XYZ Label' total neuron marker.

-For the spatial analysis scripts the outputs (single column table as a .csv) does not provide the ROI number or ?ID-XXXX) number which makes determining outliers or evaluating the output Parametric Image difficult. Please provide a second column with the relevant ROI number/ID number for clarity.

-On the documentation troubleshooting page, the link to cudnn is formatted incorrectly.

Reviewer 2

Advance summary and potential significance to field

The Gut Analysis Toolbox (GAT) by Sorensen and colleagues represents a timely and much wanted analysis workflow for enteric nervous system (ENS) researchers.

Quantification of ENS composition and structure is prone to bias because of differences in tissue processing and imaging modalities, undersampling and manual analysis methods. GAT offers a user-friendly toolkit aimed at simplifying routine image analysis procedures in ENS studies. GAT improves efficiency by facilitating unbiased analysis of larger tissue areas, multiple neurochemical markers, and various tissue samples. A number of other cell segmentation tools are available to the community but GAT seems to outperform these. The proximal-neigbour analysis option is a smart addition that helps to standardize the quantification of enteric neuron density.

Comments for the author

The authors mention that GAT (eg. in the abstract) can capture the variability associated with the differences in animal species, among others variables.

However, it seems that ENS images from species other than mouse were only used to train the deep learning models? Can the authors also demonstrate the performance of GAT on, for example, images of the human myenteric plexus?

Can the authors show some quantification results of the ganglia segmentation (numbers of ganglia and neurons per ganglion) by GAT (both are mentioned in Figure 1)? How is a ganglion actually defined? Besides the color-coded images in Figure 3F, most of the myenteric plexus pictures shown in the manuscript don't contain ganglia of which the borders can be easily observed. Also, how does this work in preparations of the small intestine where the borders of ganglia are even less clear.

I am not sure if the calcium imaging analyses workflow should be included as an additional feature in this manuscript. Calcium imaging data are not shown and the performance of GAT on calcium imaging recordings is not documented. It's therefore impossible to judge whether this tool works well, and it cannot be compared with available calcium imaging analysis software tailored for ENS recordings.

I was wondering how the segmentation was achieved to quantify neuronal density in the images shown in Figure 3A. The tdTomato reporter is not clearly delineating neuronal cell bodies here, and is present in the enteric glia network.

potential extensions:

Have the authors tested GAT to quantify enteric glial cells? They mention that it should work using Sox10 nuclear labeling.

© 2024. Published by The Company of Biologists under the terms of the Creative Commons Attribution License (https://creativecommons.org/licenses/by/4.0/).

The toolbox is compared to other image analysis workflows. It would also be nice to see how it compares, with respect to the enteric neuron numbers for instance to manual countings.

Reviewer 3

Advance summary and potential significance to field

In their manuscript, Sorensen and colleagues describe an image analysis toolbox for deep-learning based segmentation of neurons in gut samples (GAT). The authors provide widely applicable models for the segmentation of ENS neurons in gut samples, which were trained on a highly diverse dataset. GAT should simplify and accelerate the analysis of gut specimen stained for neurons. The toolbox is available via different channels (e.g. Fiji, QuPath) and is extensively documented. Training data and models are provided via Zenodo and I greatly acknowledge the effort that the authors take to promote open source and open data. The manuscript is well written and a critical discussion is provided, also elaborating on the limitations of this toolbox and deep learning in general. Although I am not an expert in the medical context, I estimate the study to be highly important to the field. Even if the provided toolbox and models are not taken up by the community (which I hope is not the case), the pretrained models can be used for transfer learning, an option that could additionally be advertised by the authors. I highly support the publication of this work. I have, however, a few minor comments that are hopefully helpful to the authors.

Comments for the author

I don't have major points to this manuscript, only minor points/suggestions that are listed in the following:

- Lines 106/107: The authors state that staining quality is essential for COUNTEN performance. This likely also applies GAT. Can the authors discuss the effect of staining on the performance of their models? Was low-quality staining data included in the training dataset?

- A cautious note might inform potential users that quality control on ground truth data of their sampled (e.g. manual segmentation and quality check using the ZeroCostDL4Mic notebook) is essential to evaluate model performance. This could e.g. be part of the section “Limitation of the DL models” or even be an entire section for its own.

- Figure 2A: The panel showing the IoU threshold of 1 appears to have the best segmentation performance, yet the F1 value for this threshold in the plot is 0. An unexperienced reader might get confused here, because these observations seem not to fit. Instead of showing an individual cell with improving segmentation, showing a larger section with increasingly missed objects for higher IoU thresholds might be less misleading.

- Figure 2B: An arrow is partially hidden behind an image panel

- Figure 2C: Asterisks are not displayed properly and p-values might be added to the section.

- Figure 2D: A statistical test would be consistent with Figure 2C.

- Lines 205/206: The authors state that the StarDist model has smoother outlines compared to the ground truth segmentation. Accuracy in cell shape prediction might be improved by increasing the number of rays for model training, although I am aware that this might limit dataset size using the free resources of Google Colab given the increase computational cost. Such an improvement is not essential for the presented study, as cell counting accuracy is high and exact cell shape might not be relevant.

- Lines 213-215: Can the authors provide the p-values for the statistical tests?

- Lines 667/668: Were the tiles resampled to a fixed image size in order to mimic different magnifications?

© 2024. Published by The Company of Biologists under the terms of the Creative Commons Attribution License (https://creativecommons.org/licenses/by/4.0/).

- Line 730: Remove duplicate “and subsequently averaged”.

- Supplementary Table 1: The dataset is highly asymmetric. Did the authors test for bias towards mouse samples? Is it possible to reduce bias by augmentation to balance the training data?

- Figure 3B: There might be a wrong unit, I assume the authors analyzed neurons per mm^2 instead of μm^2 (as specified in the caption).

- Figure 3 H/I/J: Initially, I assumed that no statistical tests were performed for I/J. This was, however, the case, as stated in the main text. The authors could add the n.s. test result to the plots to avoid such confusion.

- Line 464: The authors state that GAT accounts for varying staining qualities. However, I did not find significant support for this statement in the current manuscript.

- Image pixel sizes for the different datasets might be provided to give users already a feeling if the GAT models are applicable to their data.

- While a large body of information is provided within this study, I did not find the training parameters for the StarDist model(s). I am aware that this information can be found in the model training reports shared via Zenodo. However, accessing these parameters requires downloading and localizing this information, which might be non-intuitive for novice users. To provide direct information, the authors could add the essential training parameters to the methods section of the manuscript.

- Out of curiosity: Did the authors compare their results with the VersatileFluo model provided by StarDist? Although this model is likely not trained on gut specimen, I would be interested in the performance comparison.

First revision

Author response to reviewers' comments

Response to Reviewer comments

We thank the reviewers for the valuable feedback. We have addressed as many suggestions as possible and agree these modifications have significantly improved the GAT software and manuscript.

We have provided responses to each of the comments below. The reviewers’ questions are highlighted in bold, and the response is below each question. The response to the reviewers contains three figures, which will be referred to as R1, R2, and R3.

Reviewer 1 Advance Summary and Potential Significance to Field...

Reviewer 1 Comments for the Author...

The GAT (Gut Analysis Toolbox) presented here by Sorensen et al. is of value to the community and is suitable to JCS, however, a number of revisions must be addressed before I would recommend it for publication. The article is well written and provides a strong case for why this tool is required, however when using the tool, a number of issues were encountered that I believe need to be fixed to ensure usage and adoption by non-expert users. The data presented is compelling and of acceptable standard for the journal, with clear and easy to follow figures. The inclusion of externally published datasets provides compelling use-case and quality of the tool provided.

My following comments are broken down into the manuscript, and the tool itself.

© 2024. Published by The Company of Biologists under the terms of the Creative Commons Attribution License (https://creativecommons.org/licenses/by/4.0/).

Manuscript comments

-Overall, the publication reads well with minor typos/revisions clarifications (set out below), and is well paced inducing the different possibilities of the GAT with enough information to get the hooked reader to visit the GitHub repository to download and begin exploring the tool.

-While the authors discuss two published methods for performing this kind of analysis (Cellpose & COUNTEN) comments or inclusion of other methods that have been developed for ENS image analysis such as Durand T, et al Communications Biology 2023 and/or calcium imaging of the ENS (Cavin, Jean-Baptiste et al The Journal of Physiology, 2023) should not be ignored. It would also be advantageous to compare or comment on commercial offerings beyond just Apeer, such as Imaris/Arivis/Avia as used in the paper Herath Madushani, Biomolecules 2023 on finding/counting objects within regions (some of which also include Stardist or other deep learning models).

We have added a reference to Parker et al., 2023, which uses Imaris to generate masks for human enteric neurons in 3D. They have also published their Imaris dataset in SPARC **(line 88 and 634)**.

We have mentioned the spot detection approach for detecting enteric neurons using Imaris, referencing Cavin et al., 2023 (**line 88**).

We have removed the supplementary text as it is not allowed in JCS. However, enough detail has been added to the supplementary figure legends, and the discussion section in the main body.

-A number of references don?t list the available DOI

This is fixed.

-Note that the acknowledgements, funding and author contribution sections are not provided.

The acknowledgment and funding section are now included. The Author contribution section is included in the manuscript submission portal.

Minor corrections

- line 108 - Sentence beginning "Multiple variables..." requires re-wording, shortening, or more clarification.

The text has been adjusted to:

The image quality affects the ability of image analysis techniques to accurately detect cells. This could be due to multiple factors, such as (i) poor quality of dissection of intestinal layers, which can affect antibody penetration and labeling of the cells in the ENS, (ii) specificity of the antibodies, markers, and fluorophores used during staining, and (iii) variations in sample preparation.

- line 130 - link to GitHub repository, reference link to deepImageJ is broken

The reference links to the Github repository and deepImageJ have been fixed.

- line 649 - Table S1, it would be advantageous to include Numerical aperture (the determinant of resolution) and not just magnification information.

The details are in the Image Acquisition section in Methods. We have clarified this by adding a comment under each table.

© 2024. Published by The Company of Biologists under the terms of the Creative Commons Attribution License (https://creativecommons.org/licenses/by/4.0/).

- line 185 - I would caution using the word ?significantly? when referring to a graph with I assume n=1 samples.

The text on the line (now **878**) has been adjusted and now reads as follows:

The total time taken to segment 1309 neurons (8 images) was faster using GAT compared to manual segmentation.

- Line 265 remove duplicate with

The text has been adjusted.

- Line 298 referencing panel 3A, is it not stretched or poorly stretched?

We have changed the title of panel 3A to 'unstretched' and made it consistent throughout the document.

- Line395 celltypes to cell types

The text has been corrected.

- Lines 517-528 more information on acquisition systems is required, software, stand, immersions, detector gating & type, etc

Thank you for this suggestion. We acknowledge the importance of providing a complete description of the acquisition setup. Considering the scope and focus of our study, we believe that the information we have already included in the manuscript sufficiently describes the microscopy setup used to acquire the data.

- Line 540 typo inusing

The text has been corrected.

- Line 584 typo An google?

The text has been corrected.

- Line 650 ?mentioned under? should be changed to ?mentioned for? the species?

The text has been corrected.

- Line 676 ?values.The? to ?values. The?

The text has been corrected.

- Line679 (and others) examples of CellPose / Cellpose please be consistent, note most other published references use ?Cellpose?

The text has been corrected throughout the document and is now written as Cellpose.

© 2024. Published by The Company of Biologists under the terms of the Creative Commons Attribution License (https://creativecommons.org/licenses/by/4.0/).

- Line 758 please confirm if this is tested or untested.
- Line 764 please confirm if this is tested or untested.

The softwares were all untested.

However, we have removed the supplementary text as it is not allowed in JCS. The different softwares are not covered in the main text except for Imaris.

- Line 768 please confirm if widefield were 2D single slices or 2D projections

Widefield images are a 2D image of the FOV, whereas confocal microscopy images are maximum intensity projections.

We have removed the supplementary text as it is not allowed in JCS. However, enough detail has been added to the supplementary figure legends, and the discussion section in the main body.

+Line 782, a comment or data on the observed S:N for the different fluorophores & modalities would be useful to the reader here.

Thank you for this suggestion. We have updated **Fig. S2** with panel **H**, which shows the variation in S:N qualitatively. We acknowledge the importance of this information; however, calculating these values reliably across our dataset would require more investigation, and we believe it is outside the current scope of our study.

- Figure 2 significance symbols in panel C have not rendered correctly.

The symbols have been adjusted and now should be rendered correctly and easily visible.

- Figure S1 B/C requires scale bars

Scale bars have now been included in **Figure S1**.

PLUGIN FEEDBACK

For the GAT plugin itself, I believe requires the most work and revision before this publication could be accepted as I encountered a number of bugs, crashes and issues during testing that for a non-expert user would render this plugin inoperable.

- I initially had difficulty getting GAT to install properly within my existing daily-driver Fiji instance which has a number of existing plugins and tools installed, and instead had to get it working with a fresh install, this should be at least noted in the guide.

Thank you for this critical feedback. We have added a line in the installation and configuration section of the guide to avoid this issue for future users: https://gut-analysis-toolbox.gitbook.io/docs#installation-and-configuration.

- The authors highlight that Stardist can be called from macros automatically and can be fed information from within the command, however their own tool doesn't support that functionality. I fail to see the scenario where tools such as the Analyse Neurons, wouldn't want to be batch scriptable or callable via the macro language. Such an extension I believe would be very valuable to the GAT plugin now at the time of publication.

© 2024. Published by The Company of Biologists under the terms of the Creative Commons Attribution License (https://creativecommons.org/licenses/by/4.0/).

Thank you for this valuable suggestion. We have implemented the functionality for GAT to be callable via the macro language in Fiji. This functionality is only implemented for "Analyse Neurons". A user can specify the parameters to be passed to "Analyse Neurons" within their workflow. It is documented at: https://gut-analysis-toolbox.gitbook.io/docs/2.-cell-counts-and-ganglia-analysis/batch-analysis.

- In numerous tools/scripts called from the GAT menu in Fiji, dialogs would popup (waitforuser commands) that are not clear. For example 'Check image to select the right channel' does not provide a clear indication what the right channel is? This should instead reference the relevant processing step such as Confirm which displayed channel has labelling for Ganglia Staining, Neuron Staining etc).

We have made this clearer by including more detail in the waitforuser dialog boxes. Please see example here: Link to one of the commit on Github.

- If I select the option not to open an image by providing a filepath and instead check that the image is open, an additional checkbox to not close (or to close all) outputs would be nice. In your worked example video on the 2nd module of the documentation you show creating the output via GAT> Analyse Neurons then go on to use the output which was closed by the tool? Instead if I use the open image for the plugin tool (especially for parameter determination), it could be safely assumed that once that parameter is determined, I will execute the relevant tool and thus need the file open.

We have added the option so that the userusers can decide if they want to close other images or not. Link to relevant commit on Github.

-For the tool 'Analyse Neurons (Multi-Channel).ijm' I have identified that it has some error catching requirements that need to be addressed. If I don't select the Calculate_Neuron_Subtype checkbox or Enter_channel_details_now this will cause the tool to crash (even leaving NA in the text fields results with an error: 'Array expected in line 209,'. Upon investigating the code I don?t see any checks that NA is in the Textbox. I can check the boxes listed above and it runs fine asking for the probability sliders and follows expected behaviour. My quick assumption is to put lines 189/190 into the if conditional from line 173, or provide a default array if not being used, and provide a catch that if NA is registered to ignore it.

We have added a test to check if the boxes have "NA" or if they are empty. If they are "NA" or empty, then it gives an error message. We also reorganized the code for getting channel names and numbers, so that the user can manually define it later if needed.
Link to commit on Github.

-The Test_Ganglia_Segmentation_Hu.ijm script can run with 'Image_already_open' but still requires a saved set of ROIs (and thus the script already being run). Perhaps make this clearer (and in other menus) by having an '*'for required or similar (understanding that Fiji menus do have limitations).

We have modified the filename, so it has a "^" at the start to distinguish it. We have also added text in bold to highlight the need for having segmented cells/ROIs to run this. The "Test_Ganglia_Segmentation_Hu.ijm" macro is the only macro in commands that requires some processing to have been done before. All the other macros just require an image for testing.

Also, if no file path is given, however image is open option is selected the script crashes as 'max_proj' isn?t defined.

© 2024. Published by The Company of Biologists under the terms of the Creative Commons Attribution License (https://creativecommons.org/licenses/by/4.0/).

Thanks for this bug report. We have made the file path optional for the Test_Ganglia_Segmentation_Hu.ijm macro. If an image is open and is not selected, then the code will report an error if the file path is not provided. Link to commit on Github.

-ROIs when saved as outputs are sometimes named (Ganglia; 'Label_' or Hu ROIs; '1') or not (re)named (nNOS ROIs; e.g. '0088-0573') it would be good if they were labelled more clearly. Also providing a multi-channel jpg with the respective ROIs displayed as an overlay would be useful for each individual ROI set, for example A flattened composite image with ganglia ROIs shown, an additional flattened composite image of Neuron ROIs shown etc.

The ROI file names are now renamed and are consistent for cell types and ganglia. It follows the format: cell_type_ROIs_filename.zip. All the ROIs in each ROIManager file are renamed to list the cell_type as a prefix. For example, first ROI for cell type Hu will be Hu_1 and so on. Link to commit on Github.

We have also added an option to save flattened composite image for each cell type and ganglia. Link to commit on Github.

-Having non-unique outputs (either files or directories) can cause issues with Fiji macros. For example, if I run the analysis, open the csv and find something isn't quite right, and I need to run the macro again but don?t close the .csv output file it will crash the macro. This occurs because excel or whichever program locks the file while its open, Fiji then tries to write to that file as per the commands in the script resulting in a crash. To overcome this, including date/time or time of day in milliseconds is a common way around this.

Thank you for reporting this bug. All the macros have been updated so that GAT will issue a warning for the user to specify a custom 'suffix' at the start of the analysis so that a unique directory will be created for each analysis. This way, the files will not be rewritten and each analysis will have a unique folder. Link to an example commit on Github for "Analyze Neurons".

The Log window is also saved with the date and time at the start of the code for tracking purposes.

-When identifying ganglia within the image, often the selection would be quite noisy (and not correct) which would throw an error saying 'Max number of label reached (65535) in line 121' [or equivalent]. The error doesn't cause the macro to crash but may concern unfamiliar users. Perhaps a lower floor limit on ganglia size could be used to filter these issues?

Thank you for this bug report. This error occurs if deepImageJ does not execute and return a binary image. The code then uses the input to deepImageJ for subsequent steps, which leads to this error as it expects a label image. We have implemented two fixes.

- If the titles of the input image to deepimageJ and the output predictions are the same, an error is displayed for the user that the ganglia segmentation has not executed properly. Link to commit on Github.
- There was a lower ganglia size limit when using deepImageJ for ganglia segmentation (code), but not when using Hu expansion. We have added 200 microns as a lower floor limit for ganglia size. Link to commit on Github

Quality of Life improvements include:

-In the Help & Support menu, rather than printing results to a table that is not clickable/selectable, I would provide submenus with direct hyperlinks. This can be a number of python scripts calling the hyperlinks. E.g. import webbrowserwebbrowser.open("https://gut-analysis-toolbox.gitbook.io/docs/") Or invoking a dialog with HTML enabled if text descriptors are required

© 2024. Published by The Company of Biologists under the terms of the Creative Commons Attribution License (https://creativecommons.org/licenses/by/4.0/).

Thank you for this suggestion. We had a script parameters dialog earlier, but there were occasions where it did not display after being clicked.

We have implemented a Help & Support Menu using script parameters with hyperlinks. We have also included the option of printing to a table just in case the script parameters dialog does not work.

-The dialog for ?Enter channel number for Cell should auto-populate with the active channel number from the image, e.g. Image opens to CH1, in confirming the channel I change it to CH2, this default dialog value should be determined by the command Stack.getPosition(channel, slice, frame); which would be placed into the dialog as the default text.

Thank you for this constructive suggestion. We have implemented this improvement. The only caveat is that if two channels need to be selected, only one will be autopopulated. Link to commit on Github.

-Provide a copy of the original ROIs and then any user-updated ROIs for transparency, for example a way of determining which were deleted by the user rather than just the text information stored in the log window.

This is now implemented where the original ROIs will be saved as an ROI file with "original" in its name. Link to commit on Github.

Minor improvements/reviews

-On a few occasions using the varying tools for identifying the ganglia would select the whole image (instead of just the ganglia portion). Perhaps having a check / popup / flag to say ?It looks like the ganglia area matches the whole image area, that?s not very likely?? then allow for a different segmentation choice/confirmation.

We have implemented this for all the scripts, where if ganglia portion is >85% of the image area, then it offers the user the choice to select a different ganglia segmentation option if needed. Link to commit on Github.

-A text file with the provided example/test data should be provided to ensure non-expert users can understand which channel is which, for example filename.tif, ch1 = 'XYZ label' Ganglia, ch2 = 'XYZ Label' total neuron marker.

We have updated the sample data information to include these details: https://zenodo.org/doi/10.5281/zenodo.10590347. A link to this has also been added to the "Help and Support menu".

-For the spatial analysis scripts the outputs (single column table as a .csv) does not provide the ROI number or ?ID-XXXX) number which makes determining outliers or evaluating the output Parametric Image difficult. Please provide a second column with the relevant ROI number/ID number for clarity.

Thank you for this suggestion. The ROI numbers/IDs are now a part of the spatial analysis outputs. Link to commit on Github.

-On the documentation troubleshooting page, the link to cudnn is formatted incorrectly.

This has been fixed.

© 2024. Published by The Company of Biologists under the terms of the Creative Commons Attribution License (https://creativecommons.org/licenses/by/4.0/).

Reviewer 2 Advance Summary and Potential Significance to Field...

The Gut Analysis Toolbox (GAT) by Sorensen and colleagues represents a timely and much wanted analysis workflow for enteric nervous system (ENS) researchers. Quantification of ENS composition and structure is prone to bias because of differences in tissue processing and imaging modalities, undersampling and manual analysis methods. GAT offers a user-friendly toolkit aimed at simplifying routine image analysis procedures in ENS studies. GAT improves efficiency by facilitating unbiased analysis of larger tissue areas, multiple neurochemical markers, and various tissue samples. A number of other cell segmentation tools are available to the community but GAT seems to outperform these. The proximal-neigbour analysis option is a smart addition that helps to standardize the quantification of enteric neuron density.

Reviewer 2 Comments for the Author..

The authors mention that GAT (eg. in the abstract) can capture the variability associated with the differences in animal species, among others variables. However, it seems that ENS images from species other than mouse were only used to train the deep learning models? Can the authors also demonstrate the performance of GAT on, for example, images of the human myenteric plexus?

The performance of the models has mostly been evaluated on mouse data as the majority of studies in enteric neuroscience focus on mouse tissue, and the image data are more readily accessible. Around 19% of the enteric neuron training dataset in GAT is from human tissue. **Fig. S2J** demonstrates the performance of the GAT enteric neuron model on a public dataset from the SPARC portal. Different rescaling factors had to be applied for the widefield and confocal data (2D maximum intensity projection) to get optimal segmentation. The large variations in percentage cell count error can be explained by the larger size of the human ganglia and the 3D organization of the enteric neurons in different layers **(Fig. S2K)**. The enteric neurons overlap with each other, and it can be challenging to delineate each cell, even by eye. GAT may not work effectively in these scenarios, but having a nuclear channel (DAPI) can aid in the manual correction of neuronal segmentation within GAT as neurons have larger nuclei. The alternative is to use 3D segmentation approaches instead, either using Imaris or by training a custom Cellpose model. **Lines 628 - 637** were added, indicating the methodology and data source.

How is a ganglion actually defined? Besides the color-coded images in Figure 3F, most of the myenteric plexus pictures shown in the manuscript don't contain ganglia of which the borders can be easily observed. Also, how does this work in preparations of the small intestine where the borders of ganglia are even less clear.

The borders of the ganglion are defined by:

1. Co-distribution of pan-neuronal marker Hu with a second marker that labels neuronal or glial fibres, and
2. the presence of a gap between individual ganglia.

For the first point, the presence of neurons will define the border of the ganglion if the ganglion marker is not clear. This is demonstrated using an image from the mouse small intestine. In **Fig R1A**, the calretinin labelling in the myenteric plexus of the mouse jejunum is sparse in the ganglia, but the presence of neurons aids in demarcating the borders from the surrounding area.
For the second point, a ganglion is considered separate from another ganglion if there is an interganglionic strand or thick nerve fibre that separates them (arrowheads in **Fig R1A and R1B**) or if there is a large gap. Generally, if the gap is greater than the diameter of a single cell, then each ganglion is considered separate. The GAT ganglion model is trained on images annotated with these criteria. However, the user can choose a different approach and use the "Define ganglia using Hu" option in GAT to outline ganglia if a more objective approach is required. Both approaches reduce bias and improve reproducibility on how the ganglion borders are defined. **Lines 530 - 532** have been updated to clarify this.

© 2024. Published by The Company of Biologists under the terms of the Creative Commons Attribution License (https://creativecommons.org/licenses/by/4.0/).

To make the ganglia more visible, we have increased the brightness in **Fig 2F** and the top panel in **Fig 3G**. Additional changes could make the images too noisy or saturated.

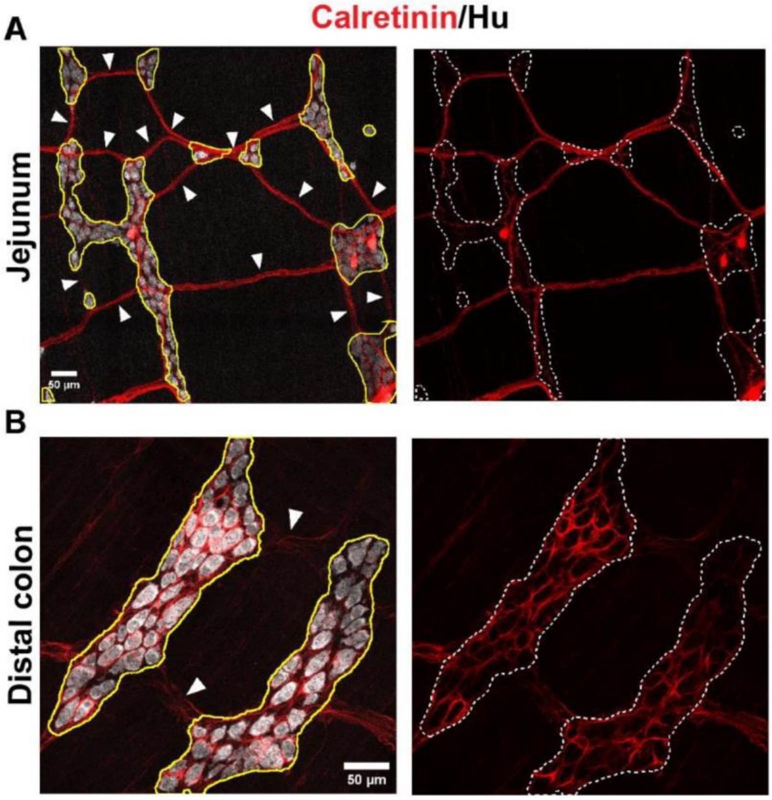

Figure R1: Defining individual ganglion within the mouse gut. *Images of the myenteric plexus of mouse jejunum (**A**) and distal colon (**B**) with calretinin and Hu labelling demonstrating how ganglionic borders are defined. The panels on the left depict the individual ganglion (yellow border) separated by interganglionic nerve fibers (white arrowheads). The panels on the right show calretinin without Hu, where the borders and the extent of each ganglion is more visible, as defined by the white dotted line.*

Can the authors show some quantification results of the ganglia segmentation (numbers of ganglia and neurons per ganglion) by GAT (both are mentioned in Figure 1)?

Neurons per ganglion quantification has been done for data in Fig. 3 but was only mentioned in the text. We have modified **lines 266 - 276** to make this clearer and have added values for the number of ganglia calculations.

We did notice a mistake in the statistical calculation for neuronal density per ganglion due to an error in how the 'mean' was calculated. This was shown as significant earlier, but the correct p-value is reported now, with the mean neuronal density counts per ganglia not being significant for DC vs PC. As the data had large outliers, the medians were compared, and it was found to be significantly different.

We have added **lines 412 – 416** discussing this.

- I am not sure if the calcium imaging analyses workflow should be included as an additional feature in this manuscript. Calcium imaging data are not shown and the performance of GAT on calcium imaging recordings is not documented. It's therefore impossible to judge whether this tool works well, and it cannot be compared with available calcium imaging analysis software tailored for ENS recordings.

Thank you for this comment.

© 2024. Published by The Company of Biologists under the terms of the Creative Commons Attribution License (https://creativecommons.org/licenses/by/4.0/).

We have removed the supplementary text as it is not allowed in JCS. However, enough detail has been added to the supplementary figure legends, and the discussion section in the main body.

I was wondering how the segmentation was achieved to quantify neuronal density in the images shown in Figure 3A. The tdTomato reporter is not clearly delineating neuronal cell bodies here, and is present in the enteric glia network.

The neurons within these images were not segmented by GAT, but manually annotated as there was no strong neuronal labelling. We only used two images for this comparison. The neurons were identified based on their low background intensities and large size, compared to enteric glia **(Fig R2 below)**. We have made this clear by adding a sentence at lines **250 – 251**. The stretched images were used to identify neurons, and the corresponding cells in the unstretched image were subsequently identified for cell counts.

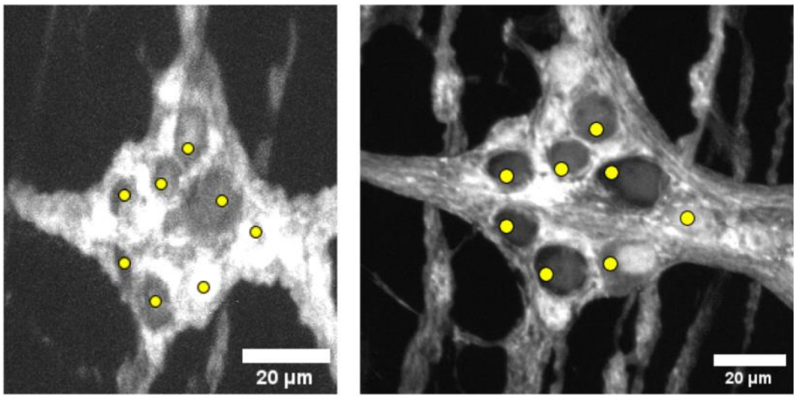

potential extensions:

- Have the authors tested GAT to quantify enteric glial cells? They mention that it should work using Sox10 nuclear labeling.

In the initial GAT release, we did have an enteric glial-specific workflow for segmenting and enumerating enteric glial cells with Sox10 labeling, which can be seen in the GitHub. However, we were not able to get as many diverse training images for Sox10 compared to Hu for enteric neurons.

Sox10 labelled enteric glial cells are round, somewhat resembling Hu labelling, but smaller in size. If we adjust the rescaling factor, to a value of e.g. 1.5 or 2, we can segment Sox10 positive cells using the enteric neuron model **(Fig R3)**. However, further finetuning and testing needs to be done. We do plan to release EGC detection and counts in an upcoming version of GAT.

- The toolbox is compared to other image analysis workflows. It would also be nice to see how it compares, with respect to the enteric neuron numbers for instance, to manual countings.

Thank you for the suggestion regarding manual count comparison. The panels in **Fig 2C and Fig 2D** show a comparison of the StarDist models to a test dataset **(lines 189 - 191)**. This test data contains 25 images of manually outlined cells. The cell count information from these manually segmented images was used to perform the count comparison. For this manuscript, we felt this comparison would be sufficient. However, in future studies, it would be beneficial to do a more extensive comparison with manual counts on larger fields of view.

© 2024. Published by The Company of Biologists under the terms of the Creative Commons Attribution License (https://creativecommons.org/licenses/by/4.0/).

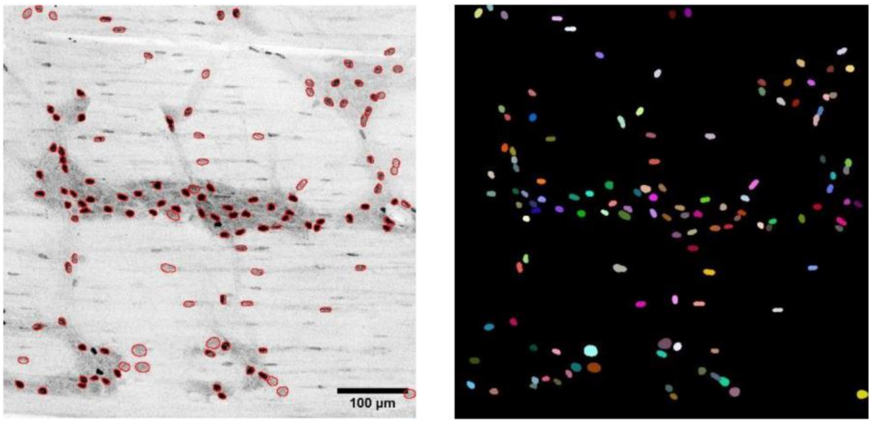

Figure R2: An example of segmented enteric glia within a myenteric wholemount from a mouse colon labelled with Sox10. The image (left) was rescaled by 1.5 and the enteric neuron model was applied using a probability of 0.5 and overlap threshold of 0.3. The segmented cells are shown as labels in the image on the right, where each cell has a unique color.

Reviewer 3 Advance Summary and Potential Significance to Field...

In their manuscript, Sorensen and colleagues describe an image analysis toolbox for deep-learning based segmentation of neurons in gut samples (GAT). The authors provide widely applicable models for the segmentation of ENS neurons in gut samples, which were trained on a highly diverse dataset. GAT should simplify and accelerate the analysis of gut specimen stained for neurons. The toolbox is available via different channels (e.g. Fiji, QuPath) and is extensively documented. Training data and models are provided via Zenodo and I greatly acknowledge the effort that the authors take to promote open source and open data. The manuscript is well written and a critical discussion is provided, also elaborating on the limitations of this toolbox and deep learning in general. Although I am not an expert in the medical context, I estimate the study to be highly important to the field. Even if the provided toolbox and models are not taken up by the community (which I hope is not the case), the pretrained models can be used for transfer learning, an option that could additionally be advertised by the authors. I highly support the publication of this work. I have, however, a few minor comments that are hopefully helpful to the authors.

Reviewer 3 Comments for the Author...

I don't have major points to this manuscript, only minor points/suggestions that are listed in the following:

- Lines 106/107: The authors state that staining quality is essential for COUNTEN performance. This likely also applies GAT. Can the authors discuss the effect of staining on the performance of their models? Was low-quality staining data included in the training dataset?

Thank you for this suggestion. Yes, we included low or varying-quality staining data in the training dataset, with some representative images in **Fig S2H**. In the earlier versions of GAT, we used models trained on images of enteric neurons that had a high signal-to-noise ratio (first image in **Fig S2H**). However, when we tested it with enteric neuron images from public data and shared the model with collaborators, we quickly realized that these models needed to be robust to variations in homogeneity and quality of immunostaining in the images. Thus, we collected a wide variety of datasets that could capture this variability in the quality of immunostaining. These include images with non-homogeneous staining, low SNR, and images from human tissue where there is increased autofluorescence.

This does not guarantee that our model will work on all enteric neuron datasets. Hence, we have made the training dataset and models available for researchers to train from scratch or finetune the models on their own data. Moreover, the flexibility to import custom segmentation during analysis allows researchers to use their preferred cell segmentation software of choice.

© 2024. Published by The Company of Biologists under the terms of the Creative Commons Attribution License (https://creativecommons.org/licenses/by/4.0/).

- A cautious note might inform potential users that quality control on ground truth data of their sampled (e.g. manual segmentation and quality check using the ZeroCostDL4Mic notebook) is essential to evaluate model performance. This could e.g. be part of the section "Limitation of the DL models" or even be an entire section for its own.

This is an important point. We have mentioned this in **lines 346**.

- Figure 2A: The panel showing the IoU threshold of 1 appears to have the best segmentation performance, yet the F1 value for this threshold in the plot is 0. An unexperienced reader might get confused here, because these observations seem not to fit. Instead of showing an individual cell with improving segmentation, showing a larger section with increasingly missed objects for higher IoU thresholds might be less misleading.

Thank you for this constructive feedback. We attempted to show a larger area with multiple cells and increasingly missed objects at higher IoU thresholds, but it still looked confusing. Instead, we have now updated **Fig 2A** to show ground truth and prediction masks with overlay at different IoU thresholds to illustrate how there are increasingly missed objects at higher IoU thresholds. Added **lines 865 – 869** in **Fig2 legend**.

- Figure 2B: An arrow is partially hidden behind an image panel

This is now fixed in **Fig 2B**.

- Figure 2C: Asterisks are not displayed properly and p-values might be added to the section. Figure 2D: A statistical test would be consistent with Figure 2C.

- Lines 213-215: Can the authors provide the p-values for the statistical tests?

We have fixed the asterisks for **Fig 2C**, and added the statistical test, showing ns for not significant in **Fig 2D**. We have also provided p-values for statistical tests throughout the manuscript.

- Lines 205/206: The authors state that the StarDist model has smoother outlines compared to the ground truth segmentation. Accuracy in cell shape prediction might be improved by increasing the number of rays for model training, although I am aware that this might limit dataset size using the free resources of Google Colab given the increase computational cost. Such an improvement is not essential for the presented study, as cell counting accuracy is high and exact cell shape might not be relevant.

Thank you for this constructive suggestion. We did increase the number of rays to 96 for model training. You are right that increasing it further could improve the cell shape accuracy, however, as you have correctly stated, we did not explore this further for this study as we were more interested in cell counts than the exact shape of the enteric neurons.

- Lines 667/668: Were the tiles resampled to a fixed image size to mimic different magnifications?

Yes, the tiles were resampled from the same image to a fixed image size to mimic different magnifications.

- Line 730: Remove duplicate "and subsequently averaged".

This has been fixed.

- Supplementary Table 1: The dataset is highly asymmetric. Did the authors test for bias towards mouse samples? Is it possible to reduce bias by augmentation to balance the training data?

Thank you for pointing this out. Yes, the training dataset for the enteric neuron model is mostly from mouse tissue (78%) due to the relative ease of data access as many enteric neuroscience labs

© 2024. Published by The Company of Biologists under the terms of the Creative Commons Attribution License (https://creativecommons.org/licenses/by/4.0/).

use mouse models for research. To mitigate this, we used augmentation during training, such as random flips, rotations, intensity changes and addition of gaussian noise, which are some of the default options provided in the StarDist Google Colab notebook. We observed that too much augmentation reduced the accuracy of segmentation, such as introducing more cell splits (data not shown).

We have added **Fig S2J**, which demonstrates the performance of the GAT enteric neuron model on a public dataset containing images of human enteric neurons from the SPARC portal. Large variations in percentage cell count error were observed, and this can be explained by the high 3D distribution of neurons in human ganglia **(Fig. S2K)**, where there is significant overlap between cells even in the 2D projection, thus making it challenging to count manually. GAT may not work effectively in these scenarios, so having a second channel such as the nuclei, may help with correcting cell counts. Alternatively, this may be better suited to 3D segmentation. **Lines 628 - 637** were added outlining the methodology.

- Figure 3B: There might be a wrong unit, I assume the authors analyzed neurons per mm^2 instead of μm^2 (as specified in the caption).

This has been fixed to show mm^2.

- Figure 3 H/I/J: Initially, I assumed that no statistical tests were performed for I/J. This was, however, the case, as stated in the main text. The authors could add the n.s. test result to the plots to avoid such confusion.

This has been fixed. **Fig 3H, I and J** now show statistical tests.

- Line 464: The authors state that GAT accounts for varying staining qualities. However, I did not find significant support for this statement in the current manuscript.

We have made this clearer by adding **Fig S2H**. These representative images of the enteric neuron model training dataset have varying staining and image qualities.

Moreover, varying staining quality could lead to either over- or under-detection of cells within GAT. Over-detection can be remedied by increasing the probability value within StarDist (accessible from GAT), which will lead to detection of lesser cell numbers. Similarly, under-detection can be fixed by decreasing the probability value to increase the numbers of cells detected.

- **Image pixel sizes for the different datasets might be provided to give users already a feeling if the GAT models are applicable to their data.**

Thank you for this question. The datasets do have varying pixel sizes, but we have normalized them by rescaling the images and have mentioned pixel sizes for rescaled training data for mouse and human neuron images **(lines 515 - 517)**. To help users determine how to accurately apply the GAT models to their data, we have information in our documentation under "Rescaling Factor" at this link: https://gut-analysis-toolbox.gitbook.io/docs/1.-choosing-the-right-parameters#id-1.1-optimising-gat-segmentation.

- **While a large body of information is provided within this study, I did not find the training parameters for the StarDist model(s). I am aware that this information can be found in the model training reports shared via Zenodo. However, accessing these parameters requires downloading and localizing this information, which might be non-intuitive for novice users. To provide direct information, the authors could add the essential training parameters to the methods section of the manuscript.**

Thank you for flagging this. We have incorporated the essential training parameters in the Methods section **(lines 550 - 576).**

© 2024. Published by The Company of Biologists under the terms of the Creative Commons Attribution License (https://creativecommons.org/licenses/by/4.0/).

- Out of curiosity: Did the authors compare their results with the VersatileFluo model provided by StarDist? Although this model is likely not trained on gut specimen, I would be interested in the performance comparison.

Thank you for this constructive suggestion. Yes, we had only done some preliminary comparisons of the GAT model with the VersatileFluo model at the start.

We still feel it would be a valuable comparison to include and have used the test data to evaluate the percentage cell count error. We found that the VersatileFluo model had a significantly higher percentage cell count error **(Fig S2I)**. Although the percentage cell count error was higher, the VersatileFluo model was still picking up the majority of neurons. It is possible that rescaling the images used could improve accuracy.

We may consider finetuning the VersatileFluo weights on GAT image data for future updates instead of training from scratch.

Second decision letter

MS ID#: JOCES/2024/261950

MS TITLE: Gut Analysis Toolbox: Automating quantitative analysis of enteric neurons

AUTHORS: Luke Sorensen, Adam Humenick, Sabrina S.B. Poon, Myat Noe Han, Narges S. Mahdavian, Matthew C Rowe, Ryan Hamnett, Estibaliz Gómez-de-Mariscal, Peter H Neckel, Ayame Saito, Keith Mutunduwe, Christie Glennan, Robert Haase, Rachel M. McQuade, Jaime P.P. Foong, Simon J.H. Brookes, Julia A. Kaltschmidt, Arrate Muñoz-Barrutia, Sebastian K. King, Nicholas A. Veldhuis, Simona E. Carbone, Daniel P. Poole, and Pradeep Rajasekhar
ARTICLE TYPE: Tools and Resources

I am happy to tell you that your manuscript has been accepted for publication in Journal of Cell Science, pending standard publication integrity checks.

Reviewer 1

Advance summary and potential significance to field

Sorensen and colleagues present a thoroughly revised and high-quality manuscript on their software tool, GAT, designed to accelerate gut tissue analysis by leveraging recent advancements in deep learning. This tool is compatible with multiple open-source and widely used image analysis software packages, making it versatile for a broad range of users.

A significant strength of their work is the inclusion of trained models from various species, researchers, and acquisition methods, which ensures the robustness of their segmentation results. The authors provide compelling evidence of a substantial reduction in analysis time compared to manual counting, and they effectively benchmark GAT against other recently published tools, demonstrating its superiority.

One of the most notable contributions of this tool is its ability to lower the barrier to entry for using advanced deep learning models through user-friendly menus and workflows. This accessibility is a major advancement for the field enabling more researchers to adopt cutting-edge techniques with greater ease.

Comments for the author

I’d like to acknowledge the large improvements made on the software tool by the authors in preparing this resubmission. The GAT toolkit is much easier to operate and less prone to errors,

© 2024. Published by The Company of Biologists under the terms of the Creative Commons Attribution License (https://creativecommons.org/licenses/by/4.0/).

crashes or issues, which aligns with the goal of it being a user-friendly tool for both experts and non-experts alike.

The authors have addressed the ability to batch process multiple files which will aid users of the tool to increase throughput and efficiencies. There have also been a number of updates to the online documentation linked to this repository which are clearer. I also didn't encounter any further issues with the plugin tool when testing it. The authors have also followed my request in providing information for non-domain experts describing the example datasets more clearly.

As for the publication itself overall, the document is improved upon from its previous submission with no identified issues or corrections. The addition of more statistical information provides clarity to the reader. The only concern I raised previously not addressed in the revised manuscript is the details of the acquisition microscopes used for the data to train the model for the GAT tool.

The authors have explained this and I can agree that beyond the information now provided it wouldn't add anything more.

Once again, I'd like to thank the authors for addressing my detailed breakdown of the tool and manuscript and recommend this is now accepted for publication.

It is also great to see the detail provided in the documentation and availability of raw data for testing.

Reviewer 2

Advance summary and potential significance to field

The authors have addressed all my remarks.

Comments for the author

I have no further comments and support publication of this paper in JCS.

Reviewer 3

Advance summary and potential significance to field

GAT streamlines and accelerates the analysis of gut samples for enteric neurons. The toolbox, which is available via FIJI, QuPath and Python is well documented and easy to use, in particular in combination with DeepImageJ. Automated image analysis reduces the bias and allows for higher statistics due to the greatly reduced analysis time. The toolbox is a valuable addition to the research field and will hopefully be widely accepted in the community.

Comments for the author

In their revised manuscript, Sorensen and coworkers addressed most reviewer comments, improved the code and extended the already comprehensive documentation. The software issues identified in the rigorous testing of Reviewer #1 were addressed, so that testing GAT on the provided data worked seamlessly for me using FIJI following the instructions given on the documentation homepage. Additional visualisations and analyses, as well as missing information (training parameters, p-values) were added to the manuscript, improving clarity and consistency. In my opinion, GAT is a well-designed toolbox that allows for streamlined, interactive and accelerated analysis of gut neurons at varying scale. I hope that the community will appreciate the efforts the authors made to create this toolbox. I fully support its publication.

© 2024. Published by The Company of Biologists under the terms of the Creative Commons Attribution License (https://creativecommons.org/licenses/by/4.0/).
